# Phylogeny, Taxonomy and Evolutionary Trade-Offs in Reproductive Traits of Gomphoid Fungi (Gomphaceae, Gomphales)

**DOI:** 10.3390/jof9060626

**Published:** 2023-05-29

**Authors:** Xue-Ping Fan, Jian-Wei Liu, Zhuliang Yang

**Affiliations:** 1 CAS Key Laboratory for Plant Diversity and Biogeography of East Asia, Kunming Institute of Botany, Chinese Academy of Sciences, Kunming 650201, China; 2 Yunnan Key Laboratory for Fungal Diversity and Green Development, Kunming 650201, China; 3 University of Chinese Academy of Sciences, Beijing 100049, China

**Keywords:** biodiversity, ectomycorrhizal fungi, evolutionary rate, fruit body size, phylogenetic analysis, spore size

## Abstract

Although functional ecology is a well-established field, our understanding of the evolutionary and ecological significance of the reproductive traits in macrofungi is still limited. Here, we reconstructed a phylogeny tree of gomphoid fungi in the narrower sense, including the species of the genera *Gomphus* and *Turbinellus* and used it to uncover the evolution of reproductive traits. Our analyses indicated that fungal fruit bodies and spores did not enlarge at a steady rate over time. Early gomphoid fungi essentially maintained their fruit body size, spore size and spore shape through the Mesozoic. In the Cenozoic, gomphoid fungi acquired significantly larger and more spherical spores by simultaneously expanding in length and width, with the fruit body size first decreasing and then enlarging. We argue that these trade-offs were driven by the effect of biological extinction and the dramatic climate changes of the Cenozoic. Gomphoid fungi initially increased in spore size and fruit body number as extinction survivors filled vacant niches. Both fruit bodies and spores eventually became larger as ecosystems saturated and competition intensified. One new species of *Gomphus* and nine new species of *Turbinellus* are described.

## 1. Introduction

Gomphoid fungi are conspicuous groups of the family Gomphaceae [1]. Systematically, the position of this group has been unclear, and the monophyly of this group has not been statistically supported [2,3,4,5]. Earlier molecular phylogenetic studies demonstrated that *Gomphus* sensu lato (*Gloeocantharellus* Singer, *Gomphus* Pers., *Phaeoclavulina* Brinkmann and *Turbinellus* Earle) and *Ramaria* sensu lato were paraphyletic within the Gomphaceae, and, consequently, a number of species published in *Gomphus* were transferred into *Gloeocantharellus*, *Phaeoclavulina* and *Turbinellus*. *Turbinellus*, once regarded as a synonym for *Gomphus* [2,6,7] and *Gomphus* s. str., have been recognized as independent genera [1,3]. However, a number of species have been published in *Gomphus* but do not have the current diagnostic morphological features of *Gomphus* s. str., causing confusion when the names of gomphoid fungi are published.

Gomphoid fungi show conspicuous diversity in the size and shape of their fruit bodies and spores. Although there is no solid evidence to demonstrate that this variation in reproductive traits is the result of an evolutionary adaptive response, previous studies [8,9,10,11] have shed light on some selective pressure from the environment on reproductive traits. Gomphoid fungi produce spores in the fruit bodies for reproduction. However, the production of these propagules is often costly; therefore, trade-offs among traits related to reproduction (e.g., between size and number of fruit bodies/spores, between reproduction and growth) become common, allowing effective reproduction at the minimal costs in a specific environment [8]. As a consequence, the reproductive syndromes that co-occur in species should be related to the trade-offs of reproductive traits in specific environments. There are commonly held beliefs that fungal species with larger fruit bodies can produce larger spores and larger spores are more spherical [12,13,14]. However, our understanding of possible correlations of fruit body size with other reproductive traits in macrofungi has been limited to current resources and environments, regardless of the evolutionary history.

Most species of gomphoid fungi are ecologically important ectomycorrhizal partners of Fagaceae, Myrtaceae and Pinaceae plants [1,15,16]. A mutualistic (ectomycorrhizal) lifestyle allows gomphoid fungi to receive carbon from their host plants and therefore adapt to predictable resources, which provides degrees of freedom for reproduction [8,17]. Large fruit bodies, which generally offer advantages by producing more spores and dispersing them farther [12], have a longer life expectancy and more sporulation events [18,19,20] and reduce the chance of desiccation and pathogens [8]. Conversely, among the advantages of small fruit bodies, are their faster maturation; thus, they have greater flexibility in adjusting their reproductive investment to fluctuating resources and climatic variation [8,9]. Likewise, large spores tend to have higher fitness and allow prolonged dormancy but are more costly to produce and more difficult to disperse than small spores [13,21,22]. If the resources a species can allocate to reproduction are limited, each species faces the challenge of either investing in a few large or more numerous small fruit bodies and likewise investing in a few large or more numerous small spores. However, how reproductive trade-offs of fungi evolved and which driving forces have shaped these trade-offs are not clear.

In this study, we sampled extensively from known gomphoid fungi and carried out a phylogenetic trait analysis in a phylogenetic context. Our aims are (1) to establish the phylogeny of gomphoid fungi and reveal their phylogenetic diversity; (2) to explore the relationships among the fruit body size, the spore size and the spore shape by incorporating information on the phylogenetic relationships of gomphoid fungi; (3) to test whether gomphoid fungi have reproductive trade-offs and to elucidate factors that might have driven these trade-offs in evolutionary history.

## 2. Materials and Methods

### 2.1. Fungal Data

Samples of the gomphoid fungi in the broader sense consisted of 101 collections from the tropical, subtropical and temperate regions of different parts of the world. In total, 108 collections representing 33 species of gomphoid fungi in the broader sense and six species of the other taxa were included in this study. Among them, 184 sequences from 71 collections were newly generated, and the remaining 58 sequences from 30 collections were retrieved from the database of GenBank (accessed on 9 March 2023). Voucher information and GenBank accession numbers for each collection are provided in Table S1. *Gomphus* is abbreviated as *G.* and *Turbinellus* as *T.*

Macroscopic characteristics were described based on field notes, photographs and literature studies. Most dried specimens were deposited in the Cryptogamic Herbarium of the Kunming Institute of Botany, Chinese Academy of Sciences (HKAS). Macromorphological measurements were conducted with a ruler in the field when the basidiome is mature and fresh. In the descriptions of pileus, some species of *Gomphus* are irregularly fan-shaped; the values of the widest part of the mature basidioma are regarded as their pileus diameter. Color codes designated in the descriptions are from Kornerup and Wanscher [23]. Microscopic features were observed and measured on dried material with light microscopy and a scanning electron microscope [24]. In the descriptions of basidiospores, the abbreviation (n/m/p) means n basidiospores measured from m basidiomata of p collections; the sizes for basidiospores are given using a range notation of the form (a–) b–c (–d): The range b–c contains a minimum of 90% of the measured values. Extreme values (a, d) are given in parentheses. Q is used to present ‘‘length/width ratio’’ of a spore in side view, and Qm is the mean Q of all basidiospores ± sample standard deviation.

Following our study and previous ones [1,9,12,13,14,25], the character data, including the maximum cap diameter as an approximation for mushroom size, the volume of basidiospores (4π/3 × (length/2) × (width/2)^2^) as an approximation for basidiospore size, and the Qm (mean of the spore length divided by width) as an approximation for basidiospore shape, were extracted for each record. Since the maximum size of the fruit body is evolutionarily conserved [5,9,12], the maximum cap diameter was used in our analyses following previous studies [9,26]. In order to obtain the maximum cap diameter of each species, 3–5 mature basidiomata per collection were measured in the field, and the maximum value was extracted. Given the skew of spore measurements within species, a 90% interval range was used in our analyses [27]. Therefore, the mean of the minimum and maximum is a reliable measure for our cross-species analysis. The mean of spore size was used for all further analyses. Some of the included taxa lacked the character data mentioned above, which were replaced with the characteristic values of the species reported in the literatures. The characteristic values are included in Table S2.

Furthermore, correlations between biological traits such as fruit body size and spore volume might be influenced by common ancestry. The lambda model in the package phytools ver. 1.2 [28] was used to detect the phylogenetic signals. Both spore volume (λ = 0.998) and fruit body size (λ = 0.869) showed strong phylogenetic signals. Then, the function “pic” [29] in the package “Ape” [30] was used to detect phylogenetic correlations between traits. The analysis showed that there was a significant phylogenetic correlation between the spore volume and the fruit body size (pr = 0.00476**). Thus, in this study, the phylogenetic spore quotient (PSQ), a measure of relative spore size compared with its value predicted by allometry and phylogeny, was used to convey how small or large a species spore is as compared to that of other species of similar fruit body size. The PSQ was calculated as Si/Sc, which corresponds to the ratio between the actual spore size (Si) and the expected spore size (Sc). To create the PSQ equation for our sample, we used the PGLS regression [31] of the log10(spore volume) vs. log10(fruit body size) to obtain Sc (the expected spore size for a fungus of its size), which is equal to 0.6307 (fruit body size) 0.7415 for our topology.

### 2.2. DNA Extraction, Amplification and Sequencing

Total genomic DNA was extracted from silica-dried material or occasionally from herbarium fragments using the Ezup Column Fungi Genomic DNA Purification Kit (Sangon Biotech, Shanghai, China) following the manufacturer’s protocol. Three nuclear markers, including the internal transcribed spacer (ITS), the translation elongation factor 1-α gene (tef1-α) and the large subunit of nuclear ribosomal RNA gene (nrLSU), were selected for the phylogenetic study. The ITS regions were amplified with primers ITS1F and ITS4 [32,33]. The nrLSU sequences were amplified with primers LROR and LR5 [34]. The tef1-α sequences were amplified with primers 983F and Efgr [35]. For some herbarium specimens with degraded DNA, the internal primers of the ITS gene 5.8S and 5.8SR and the internal primers of the tef1-α gene 1567R were employed when amplification of the larger region was unsuccessful [34,35]. All PCR conditions followed Li et al. [24]. PCR products were purified and sequenced by TSINGKE Biological Technology (Kunming, China). Sequencher ver. 4.1 (Gene Codes Corp., Ann Arbor, MI, USA) was used to assemble and edit contiguous sequences.

### 2.3. Sequence Alignment and Phylogenetic Analysis

Sequences obtained for each marker were initially aligned using MAFFT ver. 7 [36] and then manually adjusted in BioEdit ver. 2.0 [37]. The concatenated alignment is available at TreeBASE (http://purl.org/phylo/treebase/phylows/study/TB2:S30369, accessed on 11 May 2023). jModelTest ver. 0.1.1 was used to select the best-fitting likelihood model for maximum likelihood (ML) and Bayesian analyses [38]. The Akaike information criterion (AIC) was used to select among models instead of the hierarchical likelihood ratio test [39]. Model GTR+G+I was finally selected for all phylogenetic analyses.

For each marker and the simultaneous analysis of all nucleotide characters, maximum likelihood tree searches and ML bootstrapping (BS) were conducted using the web server RAxML-HPC2 on XSEDE ver. 8.2.12 on the Cipres web server [40], with 1000 rapid bootstrap analyses followed by a search for the best-scoring tree in a single run.

Bayesian inference (BI) was conducted using MrBayes ver. 3.2.7a [41]. Four Markov chain Monte Carlo chains were conducted, each beginning with a random tree and sampling one tree every 1000 generations of 10,000,000 generations. Convergence among chains was checked using Tracer ver. 1.7.1 [42], and the first 25% were discarded as burn-in to ensure that stationarity in log-likelihood had been reached. The remaining trees were used to calculate a 50% majority-rule consensus topology and posterior probabilities (PP).

### 2.4. Divergence Time Estimations

For divergence time estimation of the gomphoid fungi, we used BEAST ver. 1.10.4 [43]. For the BEAST analysis, the uncorrelated lognormal clock model was used with nucleotide substitution model GTR+G+I and birth-death speciation (uniform prior from 0 to 10 for growth rate and 0 to 1 for relative death rate; the initial value was 1 for growth and 0.5 for relative death) was specified for the dataset. Four secondary calibration ages [4] were employed in the Bayesian analysis using the normal distribution (Table S3). Two runs of four Markov chain Monte Carlo chains with 100 million generations were run with sampling every 10,000 generations. The effective sample size (ESS) of all parameters in the combined runs was >200. Log Combiner ver. 1.10.4 and TreeAnnotator ver. 1.10.4 were used to obtain a consensus tree [43]; the first 20% were considered burn-in, and the remaining 8000 trees that were used to generate maximum clade credibility tree were combined.

### 2.5. Diversification Rate Analyses

Diversification rates were estimated with BAMM ver. 2.5.0 [44] which sampled from a given time-calibrated tree using a reversible jump MCMC. The BEAST consensus species-level tree without outgroups was applied, and 20 million generations were run, sampling every 1000 generations. Prior parameters were optimized using the “setBAMMpriors” function in BAMMtools ver. 2.5.0 [45]. Post-run analysis and visualization used the BAMMtools ver. 2.5.0 in R ver. 4.1.3 [46]. Accounting for incomplete taxon sampling, the sampling fraction was specified as 0.9, representing a sampling coverage of about 90% of gomphoid species. In addition, we visually assessed the timing and tempo of diversification by constructing lineages through time (LTT) plots using the function “ltt” in the phytools ver. 1.2 [28]. One consensus tree annotated from the BEAST analysis was used to calculate the LTT plots.

### 2.6. Ancestral Character Reconstruction and Rate of Evolution

We used our phylogenetic topology to reconstruct ancestral characters and assess trends across the tree in log10(fruit body size), log10(PSQ), log10(spore size) and log10(Qm). We used BayesTraits ver. 4.0 [47] and loaded the time-calibrated tree and the character file to be traced in the “variable rates model” and the options “independent contrast” and “MCMC” with 100 million iterations and 10 million of burn-in. Upon completion of the analysis, we opened the file txt.log with Tracer ver. 1.7.1 [41] to visually verify that the analysis had reached convergence and stationarity. Another output file with the extension.txt.VarRates was processed in the program PPPostProcess [48], which allowed us to obtain a new text file used for downstream analyses.

We used the parameter ‘median scalar’ to scale the branches of the tree, so the variable rates of individual branches could be considered in the ancestral states reconstructions [48]. Then, we employed the function “anc.Bayes” in the package phytools ver. 1.2 [28], which uses the Bayesian MCMC approach to reconstruct the ancestral states of each variable on our phylogenetic trees with 20 million generations of running and 2 million of burn-in. Subsequently, we used the “plotBranchbyTrait” function in phytools ver. 1.2 to trace the rate of evolution through time. Additionally, we plotted the rate of evolution and of each trait through time using the function “ggboxplot” in the package ggplot2 ver. 3.4.0 [49].

### 2.7. Variations in Reproductive Traits through Time

The ancestral state of fruit body size, PSQ, spore size and Qm with age were obtained from “anc.Bayes” in the package phytools ver. 1.2 [28] to plot the characters changed through time by using the function “ggboxplot” in the package ggplot2 ver. 3.4.0 [49]. To plot the data through time, we binned species into 10-million-year temporal bins, which include the average of all the values for that specific temporal interval. A loop automatically classified each average age in the appropriate 10-million-year bin [50]. A column was included with the average of all bins and calculated the mean, standard deviation and total number of specimens per bin to obtain the interval of confidence per bin [50]. Additionally, the function “ggboxplot” from the package ggplot2 ver. 3.4.0 was used to visualize differences in fruit body and spore values among Mesozoic, Paleogene, Neogene and Quaternary taxon samples.

### 2.8. Phylogenetic Regressions

Phylogenetic generalized least squares (PGLS) regressions were used to determine the correlations among fruit body size, spore size, PSQ and Qm in the Mesozoic, Cenozoic and present, with associated models of Brownian motion, the Lambda model, Early Burst and Ornstein-Uhlenbeck: (a) log10(fruit body size) vs. log10(PSQ); (b) log10(fruit body size) vs. log10(spore size); (c) log10(fruit body size) vs. log10(Qm); (d) log10(spore size) vs. log10(Qm). The function “gls” and “anova” in the nlme package ver. 3.1 [51] was used to select the best model for each regression based on the lowest AIC value. Eventually, model lambda was determined for all four regressions. The package ggplot2 ver. 3.4.0 [49] was used to plot the regressions with the “ggplot” function.

## 3. Results

### 3.1. Phylogeny of Gomphoid Fungi

**Figure 1:**
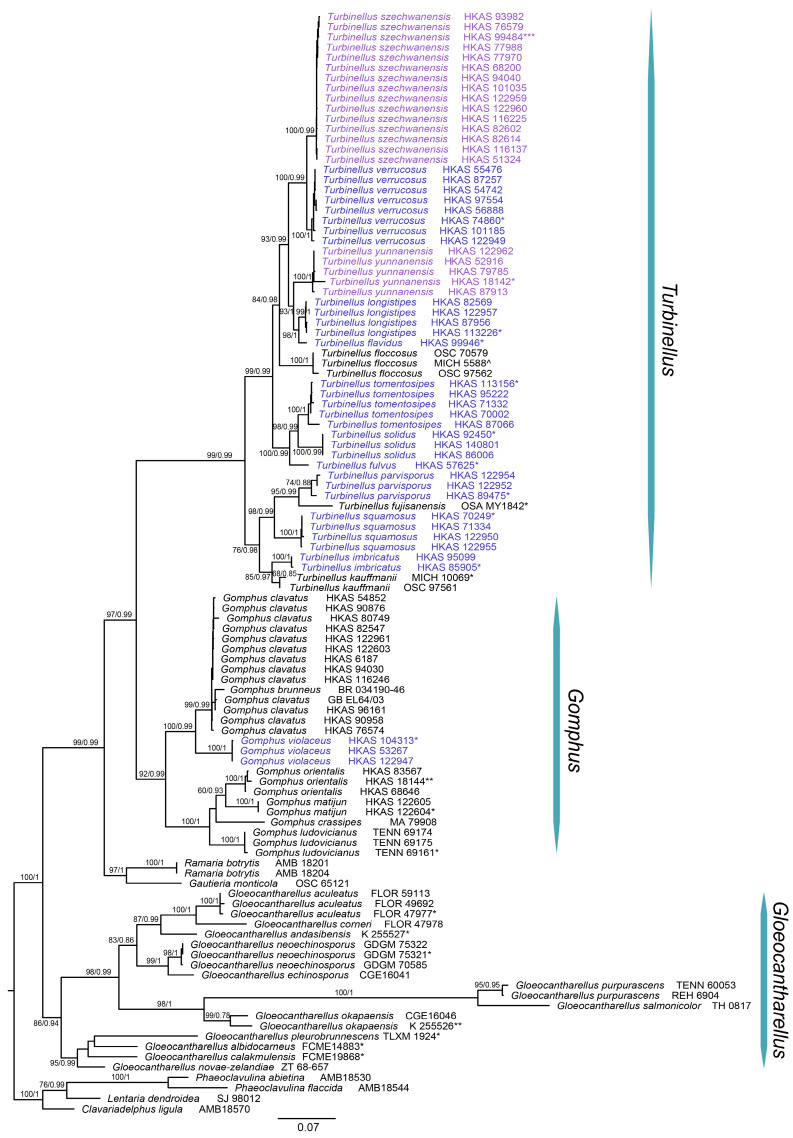
Maximum likelihood phylogeny of gomphoid fungi and related genera based on nucleotide sequences of three molecular markers (ITS, nrLSU and *tef1-α*). Bootstrap values over 50 are shown above or beneath the individual branches. Maximum likelihood bootstrap support (MLBS) and Bayesian inference posterior probability (BIPP) are on the left and right, respectively, along the branches. The new species described are indicated in blue, and new combinations described are indicated in purple. * indicates holotype specimens. ** indicates isotype specimens. *** indicates epitype specimens. ^ indicates type specimens for older name.

A total of 242 sequences from 39 species were included, of which 62 of ITS, 66 of nrLSU and 56 of tef1-α were newly generated in this study. Comparisons of three topologies from the ML analyses of the individual nuclear markers identified no well-supported conflicts. Thus, the three nuclear datasets were concatenated. The topologies of the ML and BI trees based on the concatenated datasets showed no conflicts and generally increased support values; thus, only the tree inferred from ML analysis was displayed (Figure 1).

Based on our molecular analyses (ML and BI), *Gomphus* and *Turbinellus* are each distributed in the different monophyletic clade with strong support (MLBS: 92%, BIPP: 0.99 for *Gomphus*; MLBS: 99%, BIPP: 0.99 for *Turbinellus*). The clade that combined both *Gomphus* and *Turbinellus* was resolved as a monophyletic group independent of the *Ramaria* sensu lato with strong support (MLBS: 97%, BIPP: 0.99). Furthermore, *Gloeocantharellus*, once included in gomphoid fungi in the broader sense [1], was resolved as a group paraphyletic to the gomphoid fungi in the narrower sense, including *Gomphus* and *Turbinellus*.

Our three nuclear datasets combined resolved 26 collections of *Gomphus* into five clades and one single-accession clade including seven species (with *G. brunneus* BR034190-46 clustered in the lineage of *G. clavatus*); 66 collections of *Turbinellus* into 11 clades and three single-accession clades including 14 species, of which one species of *Gomphus* and nine species of *Turbinellus* from China are putatively new to science; and two species of *Gomphus* originally described from southwestern China which should be transferred to *Turbinellus*. Finally, 15 species (including three known, two new combinations and 10 novel species described here) from China were recognized and elucidated mainly based on the phylogenetic analysis. Further morphological analyses of the related species were consistent with supporting the classification of these 10 new species and two new combinations.

### 3.2. Divergence Time Estimation

**Figure 2:**
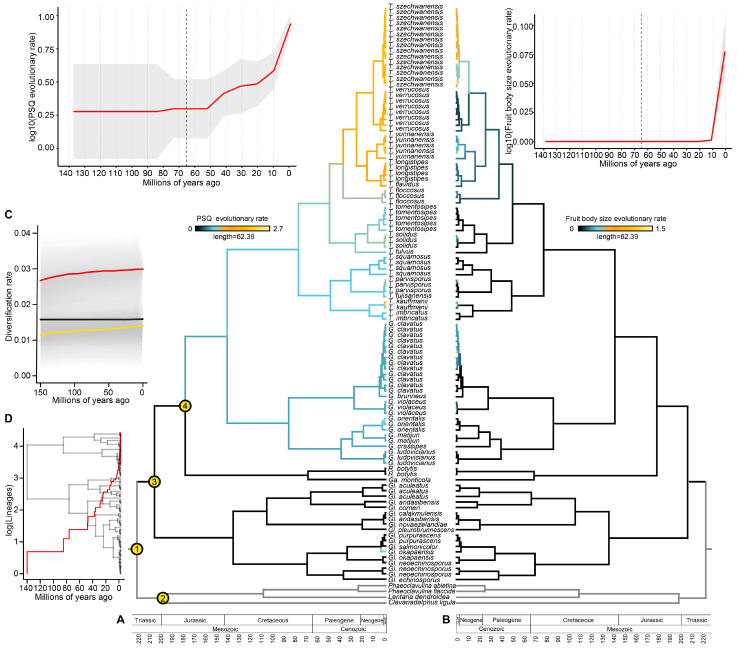
PSQ and fruit body size evolutionary change across time and phylogeny in the Mesozoic and Cenozoic. (**A**) PSQ evolutionary rate mapped onto a phylogenetic tree and log10(PSQ evolutionary rate) averaged through time per 10-million-year bins; (**B**) Fruit body size evolutionary rate mapped onto a phylogenetic tree and log10(fruit body size evolutionary rate) averaged through time per 10-million-year bins; (**C**) Rates through time for lineages with 95% credible interval according to BAMM. The red, black and yellow lines represent the speciation rate, the extinction rate and the net diversification rate, respectively; (**D**) LTT plots for a consensus tree annotated from the BEAST analysis.

The results of the BEAST analysis based on three combined nuclear genes (Figure 2A,B) showed the origin of gomphoid fungi in the narrower sense during the Lower Cretaceous about 142.85 Ma, which is consistent with Sánchez-García et al. [4]. The inferred node heights of the genus-level clades of the gomphoid fungi s. str. range from 78 Ma to 87 Ma, which places the origin of genus-level clades in the Upper Cretaceous: the *Gomphus* in the age of 87.2 Ma and *Turbinellus* in the age of 78.47 Ma. These numbers are older than some previous estimates [5,52] but later than or close to the divergence time of host plants in the families Pinaceae, Fagaceae and Myrtaceae [53,54,55,56].

### 3.3. Correlations among the Fruit Body Size, Spore Size and Spore Shape

**Figure 3:**
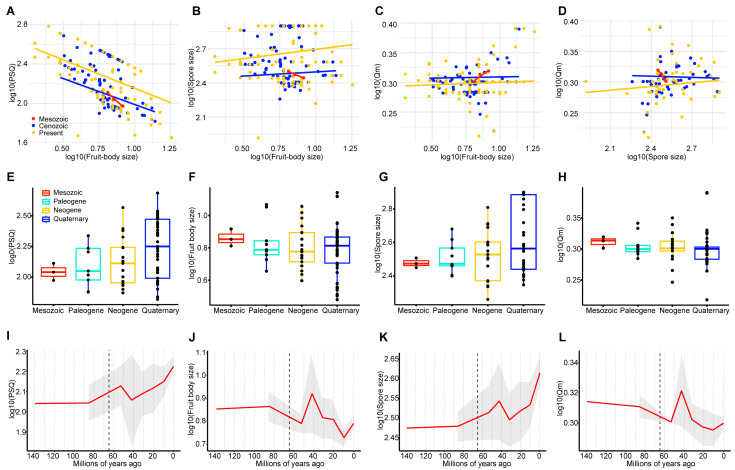
PSQ, fruit body size, spore size and Qm of Mesozoic and Cenozoic gomphoid fungi in the narrower sense. (**A**) PGLS regression of log10(PSQ versus fruit body size); (**B**) PGLS regression of log10(Spore size versus fruit body size); (**C**) PGLS regression of log10(Qm versus fruit body size); (**D**) PGLS regression of log10(Qm versus spore size); (**E**) Boxplot of log10(PSQ) for Mesozoic, Paleogene, Neogene and Quaternary; (**F**) Boxplot of log10(fruit body size) for Mesozoic, Paleogene, Neogene and Quaternary; (**G**) Boxplot of log10(Spore size) for Mesozoic, Paleogene, Neogene and Quaternary; (**H**) Boxplot of log10(Qm) for Mesozoic, Paleogene, Neogene and Quaternary; (**I**) Log10(PSQ) average through time per 10-million-year bins; (**J**) Log10(fruit body size) average through time per 10-million-year bins; (**K**) Log10(spore size) average through time per 10-million-year bins; (**L**) Log10(Qm) average through time per 10-million-year bins. Fruit body size was measured in centimeters, and spore size was measured in cubic micrometers.

PGLS analysis of reproductive traits showed that there was a significantly positive correlation between the fruit body size and the spore size of gomphoid fungi in the narrower sense in the Cenozoic (Paleogene, Neogene and Quaternary) and present but a negative correlation in the Mesozoic (Figure 3B). The correlation between the fruit body size and Qm was not significant in the Cenozoic but positive in the Mesozoic (Figure 3C). The spore size of gomphoid fungi s. str. was negatively correlated with Qm in the Mesozoic and Cenozoic (Figure 3D), indicating that larger spores were more spherical in these periods. Gomphoid species with larger fruit bodies produced larger and more spherical spores in the Cenozoic but smaller and narrower spores in the Mesozoic (Figure 3B,D).

### 3.4. Taxonomy of Gomphoid Fungi in the Narrower Sense

**Figure 4:**
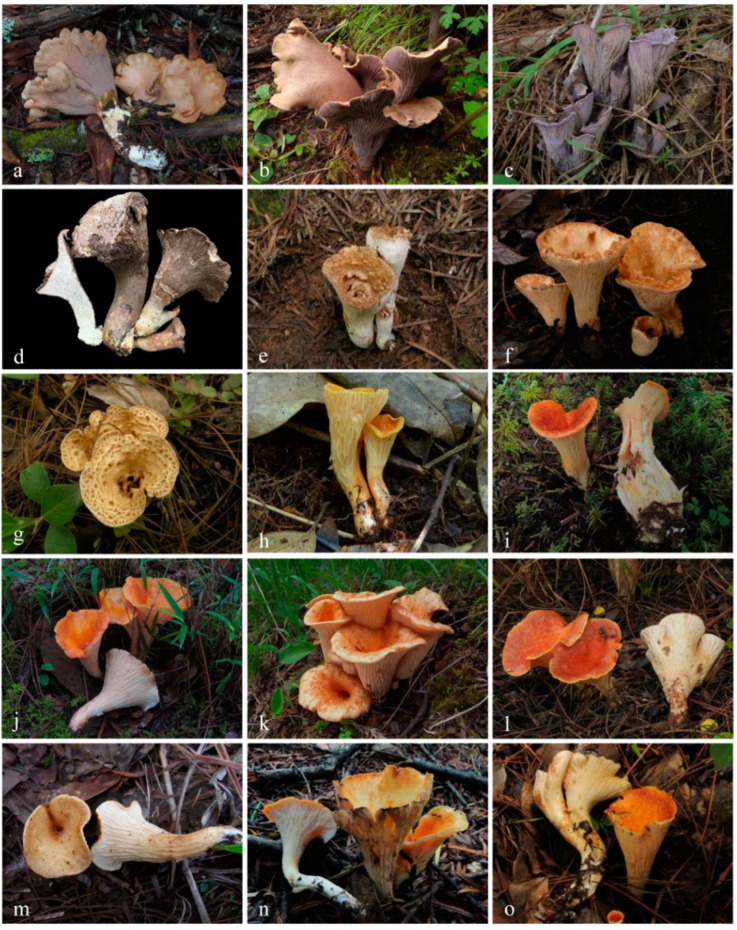
Fresh basidiomata of *Gomphus* and *Turbinellus* species. (**a**) *G. clavatus* (HKAS 122961); (**b**) *G. orientalis* (HKAS 68646); (**c**) *G. violaceus* (Type, HKAS 104313); (**d**) *G. matijun* (Type, HKAS 122604); (**e**) *T. imbricatus* (Type, HKAS 85905); (**f**) *T. parvisporus* (Type, HKAS 89475); (**g**) *T. squamosus* (Type, HKAS 70249); (**h**) *T. flavidus* (Type, HKAS 99946); (**i**) *T. longistipes* (HKAS 87956); (**j**) *T. yunnanensis* (HKAS 122962); (**k**) *T. szechwanensis* (HKAS 93982); (**l**) *T. verrucosus* (HKAS 122949); (**m**) *T. fulvus* (Type, HKAS 57625); (**n**) *T. solidus* (Type, HKAS 92450); (**o**) *T. tomentosipes* (Type, HKAS 113156).

***Gomphus clavatus*** (Pers.) Gray, Nat. Arr. Brit. Pl. (London) 1: 638 (1821). (Figure 4).

Basionym—*Merulius clavatus* Pers., Observationes Mycologicae 1:21 (1796).

Description—Basidioma up to 17 cm tall, unipileate at base and then merismatoid with 5–15 subpilei. Pileus up to 15 cm wide; irregularly fan-shaped; surface orangish brown to vinaceous brown to creamy violet; glabrous or covered with minute patches toward the crenate or undulate margin. Hymenium surface gray-violet to violet or vinaceous brown; wrinkled; irregularly reticulate to almost poroid and extending to the upper stipe. Stipe is off-white to pale violet; glabrous at apical part but tomentose to hispid toward the white base; context off-white.

Basidiospores (9–) 10–15 (–17) × (4–) 5–7.5 μm; oblong in side view; ellipsoid in ventral view; non-amyloid; surface covered with verrucose ornamentation. Basidia 60–95 × 8.5–12 μm, clavate, (2–) 4-spored. Pileipellis is composed of uninflated hyphae with pileocystidia 3–4.5 (–5.5) μm, protruding 50–120 μm from the surface. Pileus and stipe context composed of profusely interwoven and uninflated hyphae 2.5–6 µm wide. Clamp connections present in all parts of basidioma; up to 12 µm wide.

Habitat and locality—Gregarious in forests of *Abies*, *Picea*, *Pinus densata* and *Quercus semecarpifolia*. It is common in southwestern China at 2700–3600 m altitude and widely distributed in Europe and North America.

Specimens examined—AUSTRIA. Klein Walsertal: in forests with *Abies* and *Picea*, alt. 1100 m, 27 September 2016, Zhu-Liang Yang 5933 (HKAS 96161). CHINA. GANSU: Gannan Prefecture, Zhouqu County, Shatan Forest Park, in forests with *Abies*, 16 August 2012, Xue-Tai Zhu 725 (HKAS 76574). SICHUAN: Garzê Prefecture, Daofu County, Geka Township, alt. 3544 m, 17 July 2014, Jian-Wei 82 (HKAS 90876). Garzê Prefecture, Daofu County, Kongse Township, 439 km milestone of Provincial Highway 303, in forests with *Picea*, alt. 3500 m, 18 August 2013, Bang Feng 1441 (HKAS 82547). TIBET: Bomi County, alt. 2760 m, 17 July 2019, Zhu-Liang Yang 6187 (HKAS 106799). Changdu County, Gongxi Village, 7 August 2013, Kuan Zhao 324 (HKAS 80749). Gongbujiangda County, Bahe Town, in forests with *Picea*, *Pinus densata* and *Quercus semecarpifolia*, alt. 3458 m, 29 July 2014, Bang Feng 1646 (HKAS 94030). Linzhi County, Bayi District, Tunbudanggang Village, 29°39′ N, 94°44′ E, 23 July 2019, Geng-Shen Wang 532 (HKAS 116246). Linzhi County, Bujiu Township, Lamaling, 31 July 2014, Jian-Wei Liu 164 (HKAS 90958). YUNNAN: Diqing Prefecture, Xianggelila City, Haba Snow Mountain, Mianshaba, alt. 3100 m, 13 August 2003, Li-Ping Tang 621 (HKAS 54852). Diqing Prefecture, Xianggelila City, Xiaozhongdian Town, Xiaotianchi Scenic Spot, in forests of *Abies*, 9 May 2018, Jian-Wei Liu 1623 (HKAS 122603). Lijiang City, Yulong County, Alpine Garden, 22 September 2019, Jian-Wei Liu 2085 (HKAS 122961).

Notes—This species was reported from southwestern China under either the name of *G. orientalis* or *G. clavatus* [57,58]. Our molecular and morphological study of 13 collections of this group confirmed the occurrence of *G. clavatus* in southwestern China, which was often confused with *G. orientalis* (see below) due to their similar characteristics of merismatoid basidiomata, irregularly fan-shaped pileus and purplish hymenium. However, *G. clavatus* generally possesses an off-white context, smaller basidiospores (10–15 × 5–7.5 μm) and the common presence of pileocystidia in pileipellis, while *G. orientalis* has a grayish violet context, larger basidiospores (15–18 × 7–9 μm) and the rare presence of pileocystidia in pileipellis. Our phylogenetic analysis resolved *G. orientalis* as a lineage independent of *G. clavatus* (Figure 1).

***Gomphus matijun*** J.W. Liu & F.Q. Yu, Mycoscience 63: 293–297 (2022). (Figure 4).

Description—Basidioma up to 14 cm tall; unipileate to merismatoid; clavate or urceiform when young and hippocrepiform with age. Pileus up to 9 cm in diam.; gradually inflated upwards and depressed at center when mature; grayish purple; pileal margin undulate; rarely lobed. Hymenium surface grayish blue to bluish purple when young but fading to vinaceous gray or pale lilac when mature; deeply wrinkled; irregularly reticulate and extending to the stipe base. Stipe about 2–7 cm long; 1–3.5 cm in diam.; subclavate; upper part vinaceous brown or grayish purple, becoming whitish toward the base; context white to gray or slightly grayish blue.

Basidiospores [40/2/2] 9–11 (–12) × (5–) 6–7 (–8) μm; Q = (1.38–) 1.40–1.8 (–2.0); Qm = 1.65 ± 0.16, ellipsoid to oblong; non-amyloid; ornamentation verrucous. Basidia 60–90 × 7–11 μm, 1–3 spored and 2 spored common; hymenial cystidia absent. Pileipellis composed of uninflated hyphae 4–6 μm wide. Clamp connections present in all parts of basidioma.

Habitat and locality—Solitary to scattered in subalpine fagaceous forests at 1100–1200 m altitude during summer and autumn. Currently known from southwestern China.

Specimens examined—CHINA. GUIZHOU: Southwest Guizhou Autonomous Prefecture, Xingyi City, bought from Xiangjiaba Market, 24°59′ N, 104°56′ E, alt. 1163 m, 19 June 2020, Jian-Wei Liu 2287 (HKAS 122604, holotypus). Southwest Guizhou Autonomous Prefecture, Xingyi City, Zerong Town, bought from Xiangjiaba Market, alt. 1170 m, 30 August 2021, Jian-Wei Liu 2407 (HKAS 122605).

Notes—This species is close to *G. ludovicianus* R.H. Petersen, J. Justice and D.P. Lewis, *G. crassipes* and *G. orientalis* (Figure 1). However, it can be distinguished from the latter three by its grayish blue to blue or bluish purple and rarely lobed pileus, smaller basidiospores (9–11 × 6–7 μm) and basidia with 1–3 sterigmata. Furthermore, they occur in different habitats: *Gomphus matijun* occurs in fagaceous forests in southwestern China (Guizhou Province); *G. ludovicianus* produces basidiomata in forests of *Carya*, *Pinus palustris* and *Quercus* in central Louisiana and southeastern Texas; *G. crassipes* in coniferous forests in Algeria, Morocco and Spain; and *G. orientalis* in forests with *Abies*, *Picea*, *Pinus* and *Betula* in southwestern China (Tibet and Yunnan).

**Figure 5:**
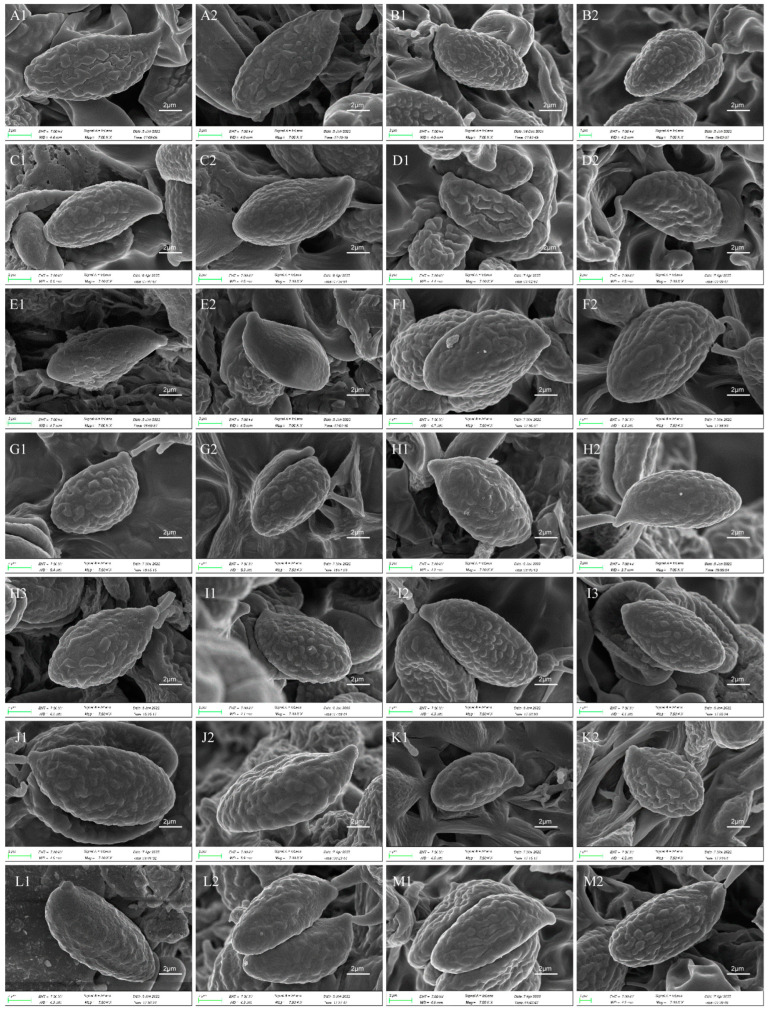
Basidiospores of *Gomphus* and *Turbinellus* species under SEM. (**A1**,**A2**) *G. orientalis* (HKAS 83567); (**B1**,**B2**) *G. violaceus* (Type, HKAS 104313); (**C1**,**C2**) *T. flavidus* (Type, HKAS 99946); (**D1**,**D2**) *T. fulvus* (Type, HKAS 57625); (**E1**,**E2**) *T. imbricatus* (Type, HKAS 85905); (**F1**,**F2**) *T. longistipes* (Type, HKAS 113226); (**G1**,**G2**) *T. parvisporus* (Type, HKAS 89475); (**H1**–**H3**) *T. solidus* (Type, HKAS 92450); (**I1**–**I3**) *T. squamosus* (Type, HKAS 70249); (**J1**,**J2**) *T. szechwanensis* (Epitype, HKAS 99484); (**K1**,**K2**) *T. tomentosipes* (Type, HKAS 113156); (**L1**,**L2**) *T. verrucosus* (Type, HKAS 74860); (**M1**,**M2**) *T. yunnanensis* (Type, HKAS 18142). Bars: (**A1**)–(**M2**) = 2 µm.

**Figure 6:**
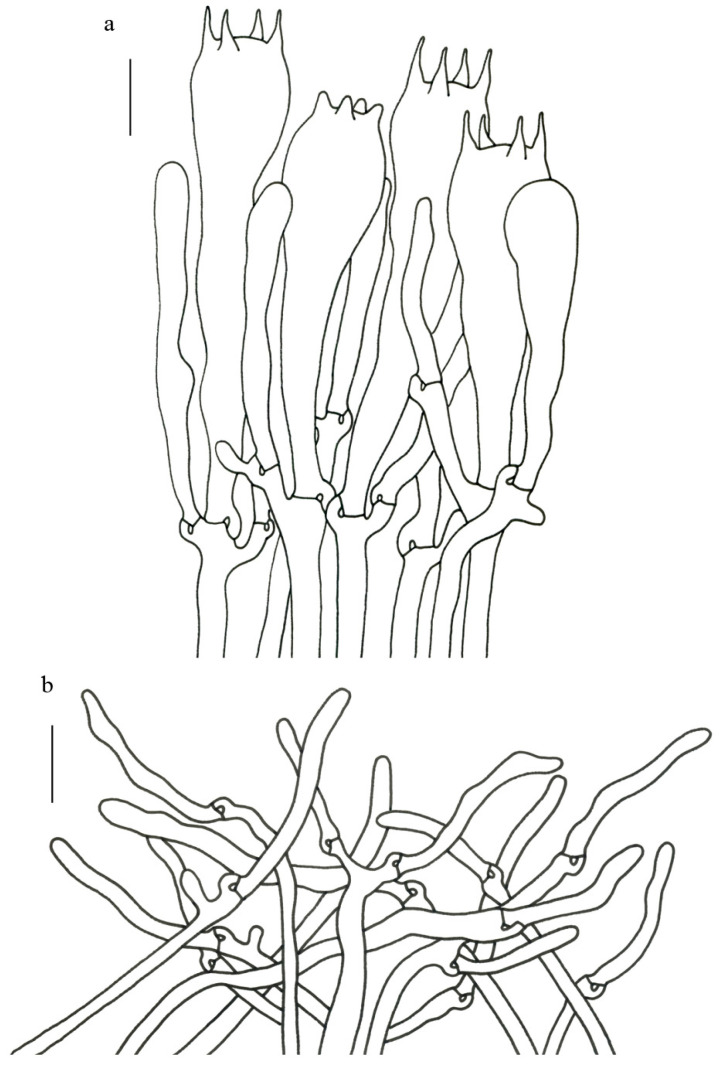
Microscopic features of *Gomphus orientalis* (HKAS 83567). (**a**) Hymenium and subhymenium; (**b**) Longitudinal section of pileipellis. Bars: (**a**)–(**b**) = 10 µm.

***Gomphus orientalis*** R.H. Petersen et M. Zang, in Zang, Li and Xi, Fungi of the Hengduan Mountains 181 (1996). (Figure 4, Figure 5 and Figure 6).

Description—Basidioma 7–13 cm tall; unipileate or merismatoid; tapering downward. Pileus 4–13 cm in diam.; lightly funnel-shaped or irregularly fan-shaped; depressed at center; surface yellowish brown (5D3) to purplish brown (8D3); pubescent; pileal margin uplifted and undulate. Hymenium surface grayish violet (14D3); deeply wrinkled; irregularly reticulate and extending longitudinally to the stipe base. Stipe about 3–5 cm long; 2–4 cm in diam.; tapering downward; solid; grayish violet (14D3); context grayish violet (14D3); unchanging on exposure; basal mycelium white (1A1).

Basidiospores [60/3/3] 15–18 (–20) × (6–) 7–9 µm; Q = (1.67–) 1.94–2.43 (–2.67); Qm = 2.18 ± 0.18; elliptic-fusiform to cylindrical and inequilateral in side view with distinct suprahilar depression; oblong in ventral view; non-amyloid; light yellowish in KOH; ornamentation strongly verrucous arranged in longitudinal ridges. Basidia 55–75 × 11–14 µm; clavate; 4-spored; sterigmata 4–7 µm long. Hymenial cystidia absent. Pileipellis composed of uninflated hyphae; usually 3–5 um wide; irregularly erect at surface; more or less parallel arranged toward pileal context; gloeoplerous hyphae abundant; yellow in water. Stipitipellis composed of some basidia-like cells and narrow hyphae (1.5–4 μm in diam.). Pileus and stipe context composed of simple-branched, narrow hyphae; usually 2–5 um wide; hyaline to yellow in KOH. Hymenial trama composed of subparallel to interwoven, undifferentiated and narrow hyphae. Clamp connections present in all parts of basidioma.

Habitat and distribution—Scattered in forests of *Abies*, *Picea*, *Pinus* and *Betula* in southwestern China (Tibet and Yunnan); fruiting from June to September between 2450 and 3400 m altitude.

Specimens examined—CHINA. YUNNAN: Lijiang City, Yulong County, Yulong Snow Mountain, in forests of *Abies* and *Picea*, alt. 3000–3400 m, 6 September 1986, R.H. Petersen 56,817 (HKAS 18144, isotype). Nujiang Prefecture, Lanping County, Tongdian Town, Luoguqing Scenic Spot, in forests with *Pinus* and *Betula*, alt. 2490 m, 16 August 2010, Bang Feng 865 (HKAS 68646). TIBET: Bomi County, near National Highway 318, in forests with *Pinus densata*, *Pinus armandii* and *Betula platyphylla*, 29°48′ N, 95°47′ E, alt. 2762 m, 30 June 2014, Qing Cai 1107 (HKAS 83567).

Notes—*Gomphus orientalis* is distinguished by the combination characters of the yellowish brown and lightly funnel-shaped or irregularly fan-shaped pubescent pileus, and the violet hymenium, stipe and context. *Gomphus orientalis* is similar and closely related to *G. matijun*. However, the former possesses a violet context, 4-spored basidia and larger basidiospores (15–18 × 7–9 µm), while the latter has a white to gray or slightly grayish blue context, generally 2-spored basidia and smaller basidiospores (9–11 × 6–7 μm). In addition, *G. orientalis* and *G. matijun* occur in different habitats: *Gomphus orientalis* is distributed in forests of *Abies*, *Picea*, *Pinus* and *Betula* at 2450–3400 m altitude, while *G. matijun* is found in fagaceous forests at 1100–1200 m altitude.

Zang et al. [59] described basidiospores of *G. orientalis* as 10.3–15.5 (–16.8) × 4.3–7.5 µm, significantly smaller than those of the isotype measured by us (15–17 × 7–9 µm), which were consistent with other two collections and were adopted here. *Gomphus orientalis* has a relative wide range of habitats. The isotype was collected in forests of *Abies* and *Picea* at 3000–3400 m altitude and the other two collections were collected in forests of *Pinus* and *Betula* at 2450–2800 m altitude. Nevertheless, these collections were treated here as the same species because molecular evidence indicated that they represented the same species (Figure 1).

**Figure 7:**
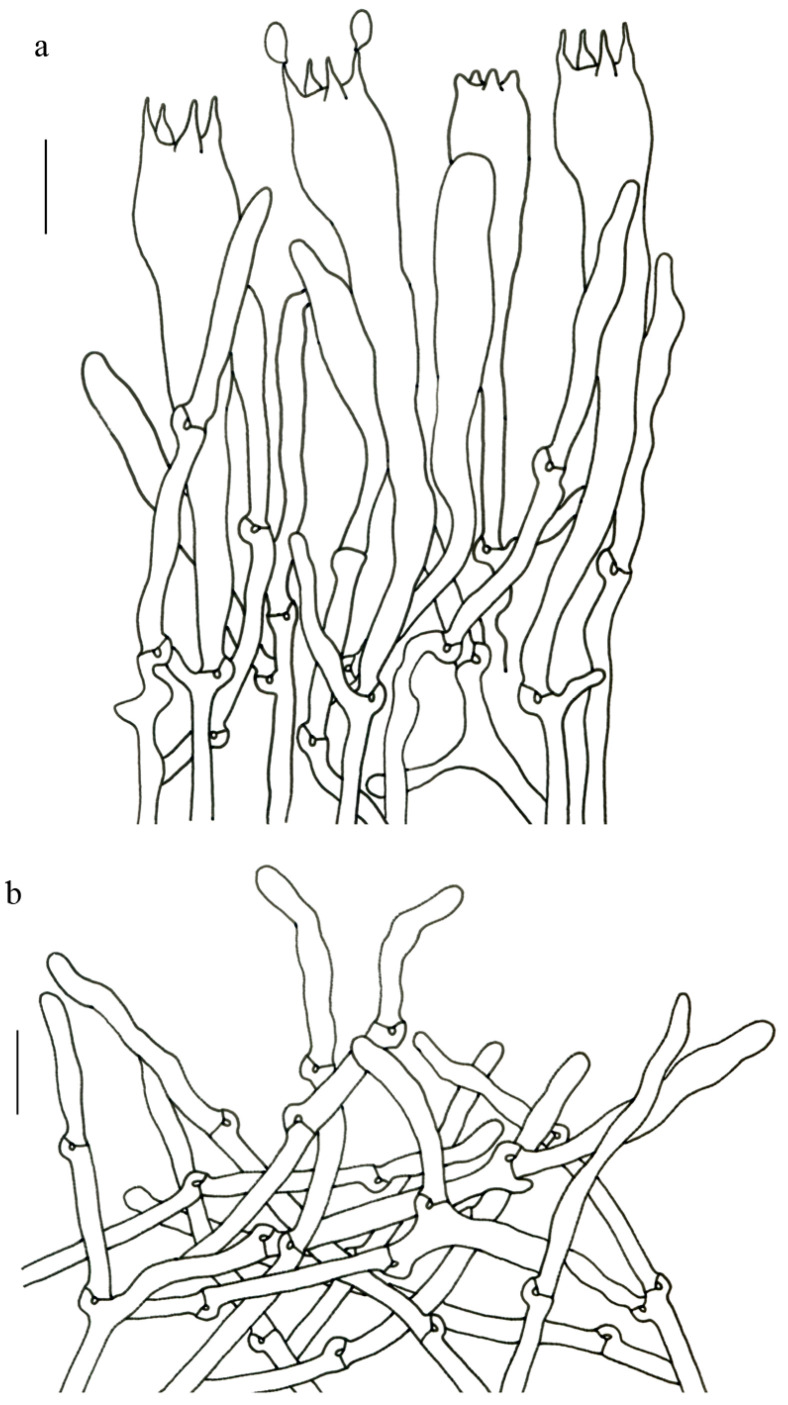
Microscopic features of *Gomphus violaceus* (Type, HKAS 104313). (**a**) Hymenium and subhymenium; (**b**) Longitudinal section of pileipellis. Bars: (**a**)–(**b**) = 10 µm.

***Gomphus violaceus*** Xue-Ping Fan & Zhu L. Yang, **sp. nov.** (Figure 4, Figure 5 and Figure 7).

MycoBank: MB 843958

Etymology—From the Latin adjective, violaceus, referring to the violet colored basidioma and context of the new species.

Holotypus—CHINA. YUNNAN: Lufeng City, Heping Town, beside Daluqing Reservoir, in forests with *Pinus*, alt. 2000 m, 18 August 2018, Zhu-Liang Yang 6154 (HKAS 104313; Genbank OQ858428/OQ858495/OQ847591).

Diagnosis—*Gomphus violaceus* is similar and closely related to *G. clavatus*. However, the former possesses a merismatoid basidioma with 2–5 subpilei, a bluish violet pileus, a violet context and the rare presence of pileocystidia in pileipellis; the latter has a merismatoid basidioma with 5–15 subpilei, a brown or grayish purple pileus, an off-white context and the common presence of pileocystidia in pileipellis. *G. violaceus* has an allopatric distribution with *G. clavatus*, in which *G. violaceus* occurs in forests with *Pinus* in 2000–2150 m altitude, while *G. clavatus* is found in forests with *Abies* and *Picea* at 2700–3600 m altitude.

Description—Basidioma 6–13 cm tall; unipileate at base and then merismatoid with 2–5 subpilei; and two subpilei common. Pileus 2–8 cm in diam.; irregularly fan- or funnel-shaped; depressed to one side; surface bluish violet (18A5) to violet (16C4); thick in the center; thin toward the ascending; pileal margin uplifted and undulate. Hymenium surface bluish violet (18A4–B4); deeply wrinkled; irregularly reticulate to almost poroid and extending to the stipe base. Stipe about 3–5 cm long; 1–3 cm in diam.; tapering downward; dilating obconical into the pileus; solid; bluish violet (18A5) to violet (16C4) or dark violet (14D4); context bright violet (18B4); unchanging on exposure; basal mycelium white (1A1).

Basidiospores [57/3/3] (11.5–) 12–14.5 (–15) × 6–8.5 (–9) µm, Q = (1.60–) 1.64–2.17 (–2.33), Qm = 1.94 ± 0.18, oblong and inequilateral in side view with distinct suprahilar depression; ellipsoid in ventral view; non-amyloid; light yellowish in KOH; ornamentation strongly verrucose with fine warts. Basidia 50–70 × 8–11 µm; clavate; generally 4-spored; sterigmata 5–9 µm long. Hymenial cystidia absent. Pileipellis composed of uninflated hyphae with simple branches that are usually 2–4 um wide and 2–7 um wide downward; irregularly erect at surface; more or less parallel arranged toward the pileal context; gloeoplerous hyphae abundant; yellow in KOH. Stipitipellis composed of some basidia-like cells and narrow hyphae (2–5 μm in diam.). Pileus and stipe context composed of profusely parallel-arranged, narrow and uninflated hyphae; 2–5 µm wide; hyaline to yellow in KOH. Hymenial trama composed of subparallel to interwoven, undifferentiated and narrow hyphae; 2–4 µm wide. Clamp connections present in all parts of basidioma.

Habitat and distribution—Solitary to scattered in subtropical forests dominated by plants of *Pinus*; endemic to Yunnan Province, China; fruiting from June to August in 2000–2150 m altitude.

Additional specimens examined—CHINA. YUNNAN: Kunming City, Qiongzhu Temple, alt. 2047 m, 8 August 2007, Li-Ping Tang 224 (HKAS 53267). Lufeng City, Heping Town, beside Daluqing Reservoir, alt. 2129 m, 18 August 2018, Jian-Wei Liu 1156 (HKAS 122947).

Notes—*Gomphus violaceus* is easily distinguished by a simply merismatoid basidioma; a bluish violet and irregularly funnel-shaped pileus; a violet context; and small basidiospores (12–14.5 × 6–8.5 µm), some of which are shared with *G. orientalis*. However, they have different habitats with *G. violaceus* in forests with *Pinus* in 2000–2150 m altitude and *G. orientalis* in forests with *Abies*, *Picea*, *Pinus* and *Betula* in 2450–3400 m altitude.

**Figure 8:**
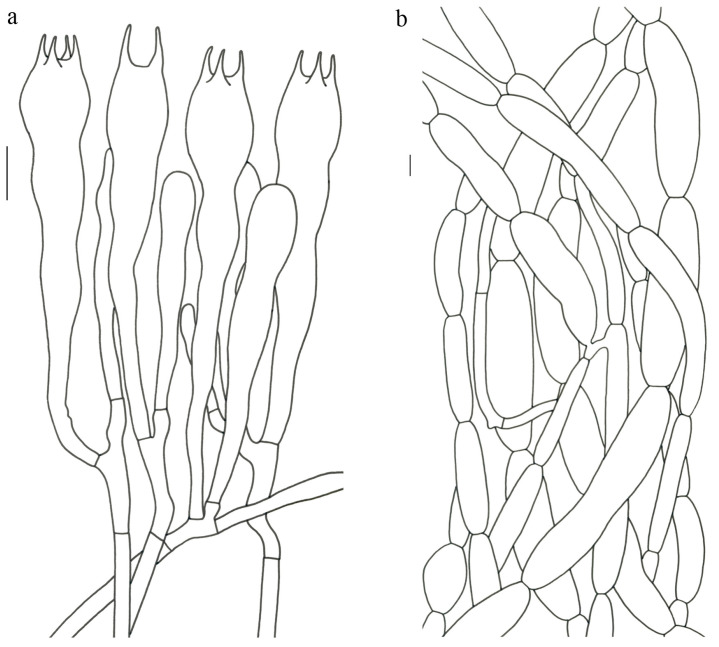
Microscopic features of *Turbinellus flavidus* (Type, HKAS 99946). (**a**) Hymenium and subhymenium; (**b**) Longitudinal section of pileipellis. Bars: (**a**)–(**b**) = 10 µm.

***Turbinellus flavidus*** Xue-Ping Fan & Zhu L. Yang, **sp. nov.** (Figure 4, Figure 5 and Figure 8).

MycoBank: MB 843946

Etymology—From the Latin adjective, flavidus, referring to the yellowish basidioma of the new species.

Holotypus—CHINA. HUNAN: Zhou City, Xuanzhang County, Mangshan Forest Park, near Zeziping, in forests of *Castanopsis*, *Pinus kwangtungensis* and *Schima superba*, alt. 1340–1460 m, 14 September 2016, Gang Wu 1826 (HKAS 99946; Genbank OQ858428/OQ858495/OQ847591).

Diagnosis—*Turbinellus flavidus* is very similar to *T. longistipes* in having sparsely scaly pileal surface, yellow hymenium, yellow cylindrical stipe and large basidiospores. However, *T. flavidus* possesses a smaller basidioma (less than 5 cm tall) with a yellow pileus, a short stipe and a yellow context, while *T. longistipes* has a larger basidioma (up to 15 cm tall) with an orange pileus, a long stipe and a white context.

Description—Basidioma 3.5–5 cm tall; unipileate; tapering downward. Pileus 0.9–2.3 cm in diam.; funnel-shaped; depressed at center; surface light yellow (3A4) to orange-yellow (4A4–6); sparsely covered with minute appressed scales; scales more or less radially arranged; pileal margin uplifted and undulate. Hymenium surface creamy (3A2–3); wrinkled; irregularly reticulate and extending to the upper stipe; sparse; reaching to margin upwards. Stipe about 1.5–1.7 cm long; 0.5–0.8 cm in diam.; approximately cylindrical; solid; orange-yellow (4A4); glabrous above the base; context pliable; thin to 0.1–0.2 cm; creamy (3A2); unchanging on exposure; basal mycelium white (1A1).

Basidiospores [22/2/1] (15.5–) 16–20 (–21) × 8–10 µm; Q = (1.82–) 1.84–2.12 (–2.21); Qm = 1.99 ± 0.1; elliptic-fusiform and inequilateral in side view with distinct suprahilar depression; ellipsoid in ventral view; non-amyloid; light yellowish in KOH; ornamentation strongly verrucose with coarse warts. Basidia 55–100 × 10–15 µm; clavate; 2–4-spored; sterigmata 5–10 µm long. Hymenial cystidia absent. Pileipellis composed of numerously inflated hyphae (7–30 μm in diam.), mostly with ampulliform swellings and little uninflated hyphae (3–6 μm in diam.); simple branched; constricted at septa; repent; more or less parallel arranged toward the pileal context; gloeoplerous hyphae abundant; yellow in water. Stipitipellis composed of some basidia-like cells; plentifully uninflated hyphae (2–6 μm in diam.) and little inflated hyphae (7–13 μm in diam.); gloeoplerous hyphae abundant; yellow in water. Pileus and stipe context composed of thin- to thick-walled hyphae; approximately parallel; hyaline to yellow in KOH. Hymenial trama composed of subparallel to interwoven, undifferentiated and narrow hyphae. Clamp connections absent in all parts of basidioma.

Habitat and distribution—In forests with *Castanopsis*, *Pinus kwangtungensis* and *Schima superba* in central China (Hunan Province) between 1300 m and 1500 m altitude.

Notes—This species almost has the smallest basidioma in the genus and is closely related to *T. tomentosipes* and *T. solidus* in our phylogenetic analysis (Figure 1). However, *T. flavidus* differs from the latter two due its smaller yellow basidioma with cream-colored context and large basidiospores. Moreover, *T. flavidus* has only been collected in forests with *Castanopsis*, *Pinus kwangtungensis* and *Schima superba* in central China, while the latter two species occur in forests with Fagaceae and *Pinus yunnanensis* in southwestern China (see below).

**Figure 9:**
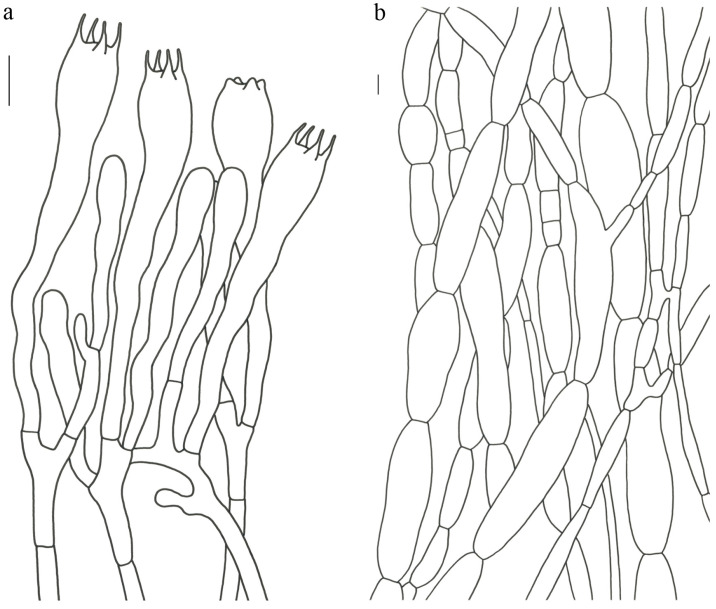
Microscopic features of *Turbinellus fulvus* (Type, HKAS 57625). (**a**) Hymenium and subhymenium; (**b**) Longitudinal section of pileipellis. Bars: (**a**)–(**b**) = 10 µm.

***Turbinellus fulvus*** Xue-Ping Fan & Zhu L. Yang, **sp. nov.** (Figure 4, Figure 5 and Figure 9).

MycoBank: MB 843949

Etymology—From the Latin adjective, fulvus, referring to the fulvous pileus and stipe of the new species.

Holotypus—CHINA. YUNNAN: Chuxiong Prefecture, Nanhua County, 15 km of Provincial Highway 217, in the mixed coniferous broad-leaved forest, alt. 2200 m, 3 August 2009, Gang Wu 93 (HKAS 57625; Genbank OQ858452/OQ858520/OQ847610).

Diagnosis—*Turbinellus fulvus* is very similar to *T. solidus* and *T. tomentosipes* in that it has a sparsely scaly pileal surface, sparse hymenium, and solid stipe. However, *T. fulvus* possesses a smaller basidioma (less than 9 cm tall), a khaki pileus, a short and pubescent stipe and smaller basidiospores (11.5–14 × 6–7 µm), while *T. tomentosipes* has a larger basidioma up to 15 cm tall, an orange-yellow pileus and a tomentose cylindrical stipe. *T. solidus* has a glabrous stipe and larger basidiospores (13.5–16 × 6.5–9 µm).

Description—Basidioma 5–9 cm tall; unipileate; tapering downward. Pileus 1.5–3.5 cm in diam.; funnel-shaped; depressed at center; surface khaki (4B3–4); sparsely covered with minute appressed scales; scales usually darker (5B5) than the pileal surface and more or less radially arranged; pileal margin extensional and undulate. Hymenium surface off-white (1A1); weakly wrinkled; longitudinal ridges extending to the stipe base and branching simply upwards toward the margin; sparse. Stipe about 3.5–4 cm long; 0.6–1.2 cm in diam.; tapering downward; dilating into the pileus; solid; khaki (5B3); covered with short pubescence; context pliable; creamy (1A2); unchanging on exposure; basal mycelium white (1A1).

Basidiospores [26/2/1] 11.5–14 × (5.5–) 6–7 (–7.5) µm; Q = 1.71–2.17 (–2.18); Qm = 1.96 ± 0.15; oblong and inequilateral in side view with distinct suprahilar depression; ellipsoid in ventral view; non-amyloid; light yellowish in KOH; ornamentation strongly verrucose arranged in longitudinal ridges. Basidia 65–85 × 10–13 µm; clavate; 4-spored; sterigmata 4–7 µm long. Hymenial cystidia absent. Pileipellis composed of profusely inflated hyphae (7–27 μm in diam.) mostly with ampulliform swellings and little uninflated hyphae (3–6 μm in diam.); simple branched; constricted at septa; repent; more or less parallel arranged toward the pileal context; gloeoplerous hyphae abundant; hyaline to yellow in water. Stipitipellis composed of some basidia-like cells; plentifully uninflated hyphae (2–6 μm in diam.) and some inflated hyphae (7–12 μm in diam.); gloeoplerous hyphae abundant; yellow in KOH. Pileus and stipe context composed of simple branched hyphae; approximately parallel; hyaline to yellow in KOH. Hymenial trama composed of subparallel to interwoven, undifferentiated and narrow hyphae. Clamp connections absent in all parts of basidioma.

Habitat and distribution—In the mixed coniferous broad-leaved forests; fruiting from June to August in southwestern China (Yunnan Province) at about 2200 m altitude.

**Figure 10:**
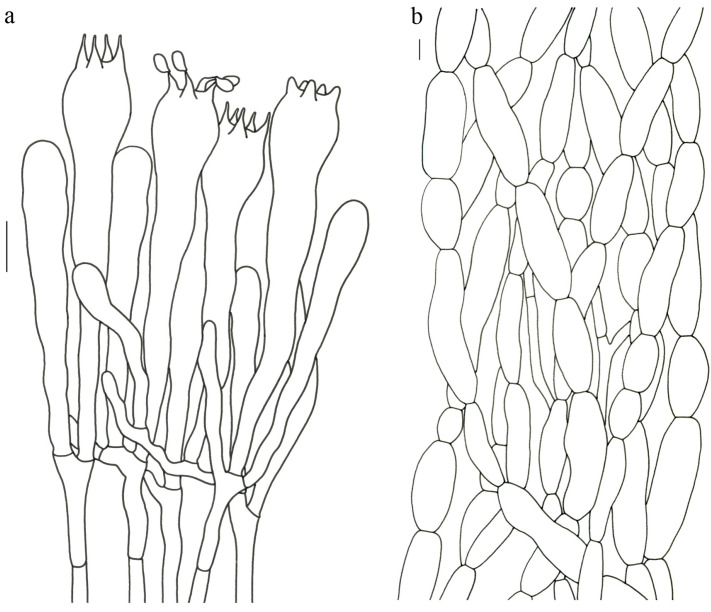
Microscopic features of *Turbinellus imbricatus* (Type, HKAS 85905). (**a**) Hymenium and subhymenium; (**b**) Longitudinal section of pileipellis. Bars: (**a**)–(**b**) = 10 µm.

***Turbinellus imbricatus*** Xue-Ping Fan & Zhu L. Yang, **sp. nov.** (Figure 4, Figure 5 and Figure 10).

MycoBank: MB 843959

Etymology—From the Latin adjective, imbricatus, referring to the pileal surface of the new species densely covered with large scales.

Holotypus—CHINA. YUNNAN: Wenshan Prefecture, Guangnan County, Nanping Town, Shuigou Mountain, in forests with fagaceous trees, 23°45′ N, 105°12′ E, alt. 1323 m, 1 August 2014, Jing Li 81 (HKAS 85905; Genbank OQ858443/OQ858509/OQ847603).

Diagnosis—*Turbinellus imbricatus* is similar to *T. kauffmanii* in having large scales on the pileal surface, but the former has a smaller basidioma less than 11 cm, a narrower pileus of less than 7 cm, a short and thin stipe (1.8–4.1 × 0.8–1.5 cm), a 4- or 5-spored basidia and the common presence of ampulliform hyphae in pileipellis. The latter has a larger basidioma up to 40 cm, a broader pileus up to 35 cm, a long and stout stipe up to 15 × 6 cm, a 2- or 4-spored basidia and the rare presence of ampulliform hyphae in pileipellis [1]. Moreover, *T. imbricatus* occurs in fagaceous forests only known to southwestern China, while *T. kauffmanii* grows in coniferous forests only known to North America.

Description—Basidioma 6–11 cm tall; unipileate; tapering downward. Pileus 1–7 cm in diam.; subcylindrical when young and narrow funnel-shaped with age; depressed at center; surface light brown (5B4); radial bulge of the surface densely covered with large scales; scales with radial stripes and rolling up in waves; pileal margin extending over hymenium and undulate. Hymenium surface off-white (1A1); slightly wrinkled; irregularly reticulate and extending to the upper stipe; sparse; thin to 0.2–0.4 cm. Stipe about 1.8–4.1 cm long; 0.8–1.5 cm in diam.; tapering downward; dilating obconical into the pileus; solid; white (1A1); glabrous above the base; context pliable; thin to 0.1–0.2 cm; white (1A1); unchanging on exposure; basal mycelium white (1A1).

Basidiospores [40/2/2] 11.5–14 × 6–8 µm; Q = 1.71–2.15 (–2.17); Qm = 1.91 ± 0.13; oblong and inequilateral in side view with distinct suprahilar depression; ellipsoid in ventral view; non-amyloid; light yellowish in KOH; ornamentation slightly verrucose with fine warts. Basidia 60–85 × 9–14 µm; clavate; generally 4- or 5-spored; sterigmata 4.5–9 µm long. Hymenial cystidia absent. Pileipellis composed of profusely inflated hyphae (7–20 μm in diam.), mostly with ampulliform swellings and barely uninflated hyphae (2–6 μm in diam.); simple branched; constricted at septa; repent; more or less parallel arranged toward the pileal context; gloeoplerous hyphae abundant; yellow in KOH. Stipitipellis composed of some basidia-like cells and profusely uninflated hyphae (2–8 μm in diam.); gloeoplerous hyphae abundant; yellow in water. Pileus and stipe context composed of thin- to thick-walled hyphae; approximately parallel; hyaline to yellow in KOH. Hymenial trama composed of subparallel to interwoven, undifferentiated, thin-walled and narrow hyphae. Clamp connections absent in all parts of basidioma.

Habitat and distribution—In mingled forests dominated by fagaceous plants; fruiting from June to August in southwestern China (Yunnan Province) in 1300–1400 m altitude.

Additional specimen examined—CHINA. YUNNAN: Wenshan Prefecture, Guangnan County, Nanping Town, Shuigou Mountain, in mingled forest dominated by fagaceous plants, 23°40′ N, 105°12′ E, alt. 1398 m, 1 August 2014, Pan-Meng Wang 18 (HKAS 95099).

**Figure 11:**
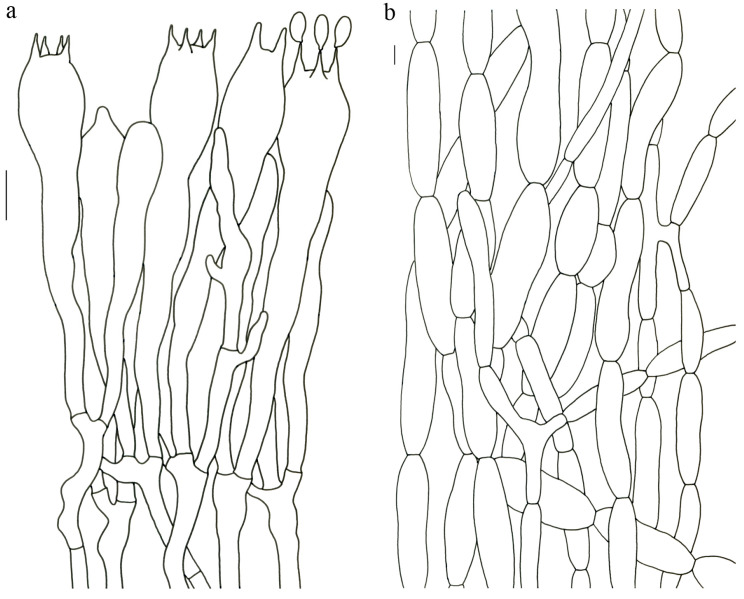
Microscopic features of *Turbinellus longistipes* (Type, HKAS 113226). (**a**) Hymenium and subhymenium; (**b**) Longitudinal section of pileipellis. Bars: (**a**)–(**b**) = 10 µm.

***Turbinellus longistipes*** Xue-Ping Fan & Zhu L. Yang, **sp. nov.** (Figure 4, Figure 5 and Figure 11).

MycoBank: MB 843960

Etymology—From the Latin longistipes, referring to the long stipe of the new species.

Holotypus—CHINA. YUNNAN: Lijiang City, Yulong County, 99 Longtan Scenic Spot, alt. 3600 m, 27 August 2020, Fei-Fei Liu 316 (HKAS 113226; Genbank OQ858464/OQ858533/OQ847621).

Diagnosis—*Turbinellus longistipes* is very similar to *T. yunnanensis* in having orange pileus with minute appressed scales. However, the former possesses a creamy to light orange basidioma, a long stipe (5–10 cm) with the slightly swollen base and larger basidiospores (15–20 × 7–9.5 µm). The latter has a reddish basidioma, a cylindrical and short stipe (2–4 cm) and smaller basidiospores (13–17 × 6.5–7.5 µm). Ecologically, *T. longistipes* occurs at 3200–3600 m altitude, while *T. yunnanensis* is distributed at 2800–3200 m altitude (see below).

Description—Basidioma 4–15 cm tall; unipileate; tapering downward. Pileus 2–10 cm in diam.; funnel-shaped; depressed at center; surface orange (5A7–6A7); sparsely covered with minute appressed scales; pileal margin undulate or entire. Hymenium surface creamy (2A2) to creamy orange (5A2); wrinkled; irregularly reticulate and extending to the upper stipe; sparse; thin to 0.2 cm. Stipe 5–10 cm long; 0.5–1 cm in diam.; subcylindrical; base slightly swollen; nearly solid; creamy (1A2–3) to creamy orange (5A2); glabrous; context pliable; thick to 0.2–0.4 cm; white (1A1); unchanging on exposure; basal mycelium white (1A1).

Basidiospores [82/4/4] 15–20 (–21) × 7–9.5 (–10) µm; Q = (1.76–) 1.82–2.33 (–2.5); Qm = 2.06 ± 0.16; oblong to cylindrical in side view with distinct suprahilar depression; ellipsoid in ventral view; non-amyloid; light yellowish in KOH; ornamentation strongly verrucose arranged in longitudinal ridges. Basidia 65–120 × 11–20 µm; clavate, 2–4-spored; sterigmata 5–10 µm long. Hymenial cystidia absent. Pileipellis composed of profusely inflated hyphae (7–24 μm in diam.), mostly with ampulliform swellings and little uninflated hyphae (3–6 μm in diam.); simple branched; constricted at septa; repent; more or less parallel arranged toward the pileal context; gloeoplerous hyphae abundant; hyaline to yellow in KOH. Stipitipellis composed of some basidia-like cells, inflated hyphae (7–18 μm in diam.) and uninflated hyphae (2–6 μm in diam.); gloeoplerous hyphae abundant; yellow in KOH. Pileus and stipe context composed of thin- to thick-walled hyphae; approximately parallel; hyaline to yellow in KOH. Hymenial trama composed of subparallel to interwoven, undifferentiated and narrow hyphae. Clamp connections absent in all parts of basidioma.

Habitat and distribution—In subtropical forests dominated by plants of *Abies*, *Picea*, *Pinus densata* and *Quercus semicarpifolia*; fruiting from June to September in southwestern China (Yunnan Province) between 3200 and 3600 m altitude.

Additional specimens examined—CHINA. YUNNAN: Diqing Prefecture, Xianggelila City, Xiaozhongdian Town, in forests with *Picea* and *Abies*, alt. 3300 m, 21 August 2013, Qi Zhao 2190 (HKAS 87956). Lijiang City, Yulong County, Yuhu Reservoir, in forests with *Pinus densata* and *Quercus semicarpifolia*, alt. 3200 m, 30 August 2013, Bang Feng 1463 (HKAS 82569). Nujiang Prefecture, Lanping County, Tongdian Town, 1 September 2018, Jian-Wei Liu 1472 (HKAS 122957).

Notes—*Turbinellus longistipes* is easily distinguished by its creamy to light orange and large basidioma, long stipe (5–10 cm) with slightly swollen base and large basidiospores. This species may also be confused with *T. verrucosus* in the wild. However, *T. longistipes* differs from *T. verrucosus* by its minute appressed scales sparsely covering the pileal surface and long stipe with slightly swollen base (see below).

**Figure 12:**
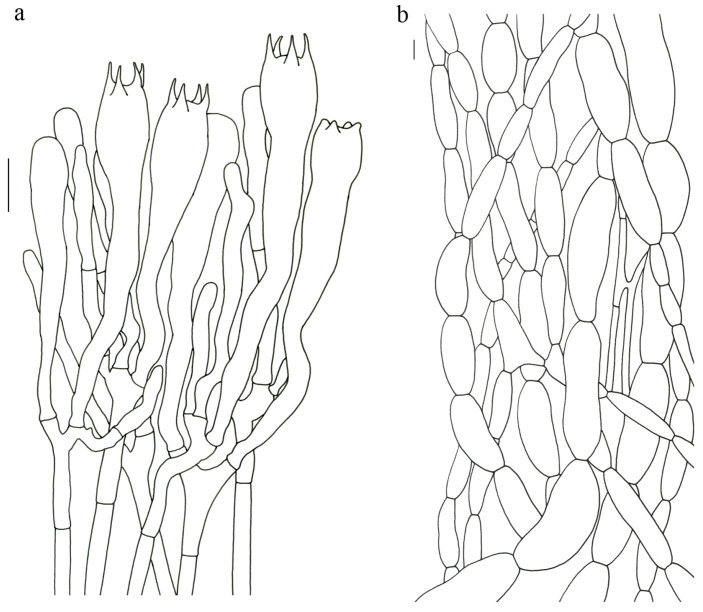
Microscopic features of *Turbinellus parvisporus* (Type, HKAS 89475). (**a**) Hymenium and subhymenium; (**b**) Longitudinal section of pileipellis. Bars: (**a**)–(**b**) = 10 µm.

***Turbinellus parvisporus*** Xue-Ping Fan & Zhu L. Yang, **sp. nov.** (Figure 4, Figure 5 and Figure 12).

MycoBank: MB 843961

Etymology—From the Latin prefix, parvi-, and the Latin suffix, -sporus, referring to the small basidiospores of the new species.

Holotypus—CHINA. YUNNAN: Kunming City, Panlong District, Wild Duck Lake Park, alt. 2000–2200 m, 18 August 2012, Yan-Chun Li 2832 (HKAS 89475; Genbank OQ858450/OQ858516/OQ847607).

Diagnosis—*Turbinellus parvisporus* is similar to *T. fujisanensis* in having large deltoid scales on pileal surface and small basidiospores, but the former has a smaller basidioma less than 9 cm tall, with the reticulation of hymenium becoming stronger upwards and the common presence of inflated hyphae (7–24 μm in diam.) in pileipellis and stipitipellis. The latter has a larger basidioma up to 15 cm tall, an uniformly forked hymenium and the rare presence of inflated hyphae in all parts of the basidioma.

Description—Basidioma 4–9 cm tall; unipileate; tapering downward. Pileus 3–7 cm in diam.; funnel-shaped; depressed at center; surface flesh-colored (6A4–5); radial bulge of the surface densely covered with large deltoid scales especially in the center; scales rolling up in waves; pileal margin uplifted and undulate. Hymenium surface white (1A1) to creamy (3A2); strongly wrinkled; irregularly reticulate and extending to the upper stipe. Stipe about 1.5–3.5 cm long; 0.6–1.2 cm in diam.; tapering downward; dilating obconical into the pileus; hollow; khakis (4B3) to flesh-colored (6A5); glabrous above the base; context pliable; thin to 0.1–0.2 cm; white (1A1); unchanging on exposure; basal mycelium white (1A1).

Basidiospores [41/3/2] 10–13 × 5.5–7 µm, Q = 1.43–2.09 (–2.18); Qm = 1.78 ± 0.2, ellipsoid to oblong and inequilateral in side view with distinct suprahilar depression; ellipsoid in ventral view; non-amyloid; light yellowish in KOH; ornamentation moderately verrucose with coarse warts. Basidia 50–80 × 9–13 µm; clavate; generally 4-spored; sterigmata 4–8 µm long. Hymenial cystidia absent. Pileipellis composed of profusely inflated hyphae (7–24 μm in diam.), mostly with ampulliform swellings and little uninflated hyphae (3–6 μm in diam.); simple branched; constricted at septa; repent; more or less parallel arranged toward the pileal context; gloeoplerous hyphae abundant; hyaline to yellow in KOH. Stipitipellis composed of some basidia-like cells; plenty of inflated hyphae (7–15 μm in diam.) and uninflated hyphae (2–6 μm in diam.); gloeoplerous hyphae abundant; hyaline to yellow in KOH. Pileus and stipe context composed of thin- to thick-walled hyphae; approximately parallel; hyaline to yellow in KOH. Hymenial trama composed of subparallel to interwoven, undifferentiated and narrow hyphae. Clamp connections absent in all parts of basidioma.

Habitat and distribution—On humid, heavy metal-contaminated soils; fruiting from June to August in southwestern China (Yunnan Province) between 1800 m and 2000 m altitude.

Additional specimens examined—CHINA. YUNNAN: Dali Prefecture, Heqing County, Xiyi Town, Beiya Village, 27 August 2018, Jian-Wei Liu 1241 (HKAS 122952). Dali Prefecture, Heqing County, Xiyi Town, Beiya Village, alt. 1800–1900 m, 18 August 2018, Jian-Wei Liu 1263 (HKAS 122954).

**Figure 13:**
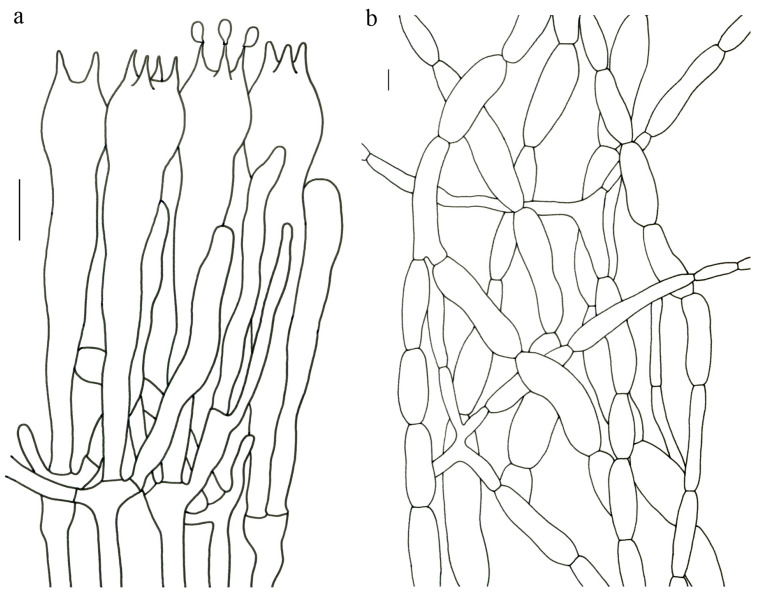
Microscopic features of *Turbinellus solidus* (Type, HKAS 92450). (**a**) Hymenium and subhymenium; (**b**) Longitudinal section of pileipellis. Bars: (**a**)–(**b**) = 10 µm.

***Turbinellus solidus*** Xue-Ping Fan & Zhu L. Yang, **sp. nov.** (Figure 4, Figure 5 and Figure 13).

MycoBank: MB 843963

Etymology—From the Latin adjective, solidus, referring to the solid stipe of the new species.

Holotypus—CHINA. YUNNAN: Wenshan Prefecture, Guangnan County, Nanping Town, in forests with *Keteleeria fortunei* and *Pinus yunnanensis*, 23°45′ N, 105°12′ E, alt. 1350 m, 7 August 2015, Kuan Zhao 820 (HKAS 92450; Genbank OQ858517/OQ847608).

Diagnosis—*Turbinellus solidus* is similar to *T. yunnanensis* due to their small basidiomata with the orange-yellow to orange and scaly pileal surface and medium-sized basidiospores. However, the former possesses a white to creamy basidioma and a solid stipe, while the latter has a reddish basidioma and a hollow stipe. Ecologically, *T. solidus* is distributed in subtropical forests dominated by Fagaceae, *Keteleeria fortunei* and *Pinus yunnanensis* at 1300–1600 m altitude, while *T. yunnanensis* occurs in forests of *Abies*, *Picea*, *Pinus densata* and *Quercus* at 2800–3200 m altitude (see below).

Description—Basidioma 6–10 cm tall; unipileate; tapering downward. Pileus 1.8–7.5 cm in diam.; funnel-shaped; depressed at center; cup depth 3–6 cm; surface orange-yellow (4A3–4) to orange (6A7–8); sparsely covered with minute appressed scales; scales more or less radially arranged; pileal margin uplifted and undulate. Hymenium surface off-white (1A1) to creamy (3A2); wrinkled; irregularly reticulate and extending longitudinally to the upper stipe; sparse; thin to 0.2 cm. Stipe about 2–5.5 cm long; 0.5–1.5 cm in diam.; tapering downward; dilating obconically into the pileus; solid; white (1A1) to creamy (1A2); glabrous above the base; context pliable; thin to 0.1–0.2 cm; white (1A1); unchanging on exposure; basal mycelium white (1A1).

Basidiospores [59/3/3] (12–) 13.5–16 (–17.5) × (6–) 6.5–9 µm; Q = (1.67–) 1.75–2.22 (–2.33); Qm = 1.96 ± 0.15; oblong and inequilateral in side view with distinct suprahilar depression; ellipsoid in ventral view; non-amyloid; light yellowish in KOH; ornamentation moderately verrucose with coarse warts. Basidia 50–90 × 9–14 µm; clavate; 2–4-spored; sterigmata 4–9 µm long. Hymenial cystidia absent. Pileipellis composed of profusely inflated hyphae (7–20 μm in diam.), mostly with ampulliform swellings and little uninflated hyphae (3–6 μm in diam.); simple branched; constricted at septa; repent; more or less parallel arranged toward the pileal context; gloeoplerous hyphae abundant; yellow in water. Stipitipellis composed of some basidia-like cells; plenty of inflated hyphae (7–15 μm in diam.) and many uninflated hyphae (2–6 μm in diam.); gloeoplerous hyphae abundant; hyaline to yellow in KOH. Pileus and stipe context composed of moderately thin- to thick-walled hyphae; approximately parallel; hyaline to yellow in KOH. Hymenial trama composed of subparallel to interwoven, undifferentiated, thin-walled and narrow hyphae. Clamp connections absent in all parts of basidioma.

Habitat and distribution—Scattered in subtropical forests with Fagaceae, *Keteleeria fortunei* and *Pinus yunnanensis*; on loamy and humid soils; fruiting from June to August in southwestern China (Yunnan Province) between 1300 and 1600 m altitude.

Additional specimens examined—CHINA. YUNNAN: Wenshan Prefecture, Guangnan County, Nanping Town, Shuigou Mountain, in fagaceous forests, 23°45′ N, 105°12′ E, alt. 1323 m, 1 August 2014, Jing Li 78 (HKAS 140801). Wenshan Prefecture, Qiubei County, Jinping Town, Xiangqi Village, in forests with Fagaceae and *Pinus yunnanensis*, 24°05′ N, 104°14′ E, alt. 1569 m, 10 August 2014, Jing Li 180 (HKAS 86006).

**Figure 14:**
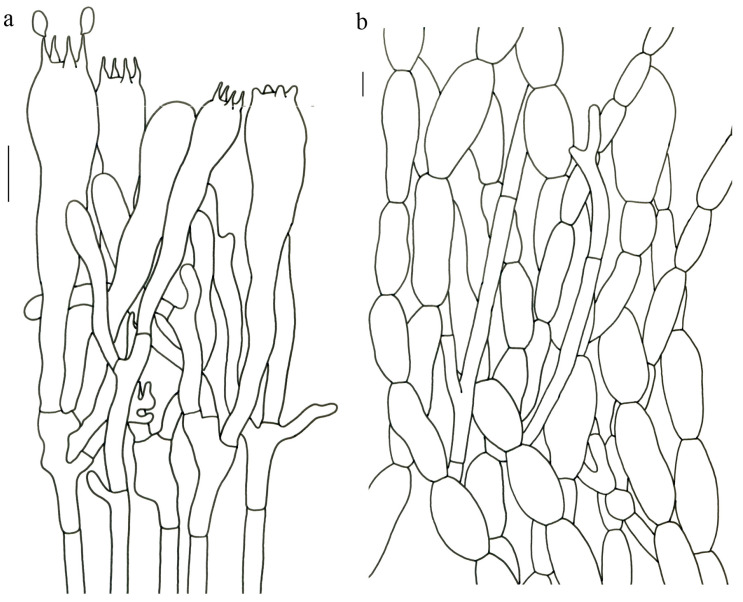
Microscopic features of *Turbinellus squamosus* (Type, HKAS 70249). (**a**) Hymenium and subhymenium; (**b**) Longitudinal section of pileipellis. Bars: (**a**)–(**b**) = 10 µm.

***Turbinellus squamosus*** Xue-Ping Fan & Zhu L. Yang, **sp. nov.** (Figure 4, Figure 5 and Figure 14).

MycoBank: MB 843964

Etymology—From the Latin adjective, squamosus, referring to the pileal surface of the new species covered with large scales.

Holotypus—CHINA. YUNNAN: Dali Prefecture, Binchuan County, Jizushan Town, Shanqiansi Village, in forests with *Pinus yunnanensis*, alt. 2000–2100 m, 11 August 2011, Qing Cai 654 (HKAS 70249; Genbank OQ858444/OQ858510/OQ847604).

Diagnosis—*Turbinellus squamosus* is very similar to *T. fujisanensis* in having large deltoid scales densely covering the pileal surface, but the former has a smaller basidioma less than 9 cm tall; a narrower pileus (1–6 cm in diam.); the reticulation of hymenium becoming stronger upwards; larger basidiospores (11.5–14.5 × 6–7 µm); and the common presence of inflated hyphae (7–25 μm in diam.) in pileipellis. The latter has a larger basidioma up to 15 cm tall, a broader pileus (5–10 cm in diam.), a uniformly forked hymenium, smaller basidiospores (9.5–11.5 × 5–6 µm) and the rare presence of inflated hyphae in all parts of the basidioma. Furthermore, *T. squamosus* occurs in forests of *Pinus yunnanensis* in southwestern China, while *T. fujisanensis* grows in mixed coniferous forests in Japan.

Description—Basidioma 4–9 cm tall; unipileate; tapering downward. Pileus 1–6 cm in diam.; fan- to funnel-shaped; depressed at center; surface creamy (2A3) to flesh-colored (6A3–4); densely covered with large triangular scales; scales rolling up in waves; often loose near the margin with small ones and then filling the center of the basidioma with large ones; darker (6B4–5) than the pileal surface; annularly arranged; pileal margin uplifted and undulate. Hymenium surface white (1A1) when young and creamy (2A3) with age; wrinkled; irregular ridges extending longitudinally to the upper stipe; thick to 0.2–0.5 cm. Stipe about 2–3 cm long; 0.8–1 cm in diam.; tapering downward; dilating obconically into the pileus; hollow; white (1A1) to creamy (2A3); glabrous above the base; context pliable; white (1A1); unchanging on exposure; basal mycelium white (1A1).

Basidiospores [80/4/4] (11–) 11.5–14.5 (–15) × 6–7 (–8) µm; Q = (1.64–) 1.78–2.33 (–2.50); Qm = 2.01 ± 0.17; oblong to cylindrical and inequilateral in side view with distinct suprahilar depression; ellipsoid in ventral view; non-amyloid; light yellowish in KOH; ornamentation strongly verrucose with fine warts. Basidia 50–105 × 7–14 µm; clavate; generally 4-spored; sterigmata 5–9 µm long. Hymenial cystidia absent. Pileipellis composed of profusely inflated hyphae (7–25 μm in diam.), mostly with ampulliform swellings and rarely uninflated hyphae (3–6 μm in diam.); simple branched; constricted at septa; repent; more or less parallel arranged toward the pileal context; gloeoplerous hyphae abundant; hyaline to yellow in KOH. Stipitipellis composed of some basidia-like cells; plenty of uninflated hyphae (2–6 μm in diam.) and little inflated hyphae (7–11 μm in diam.); gloeoplerous hyphae abundant; hyaline to yellow in KOH. Pileus and stipe context composed of thin- to thick-walled hyphae; approximately parallel; hyaline to yellow in KOH. Hymenial trama composed of subparallel to interwoven, undifferentiated and narrow hyphae. Clamp connections absent in all parts of basidioma.

Habitat and distribution—Scattered in subtropical forests mainly with *Pinus yunnanensis*; moderately common in southwestern China (Yunnan Province); fruiting in June to August between 1800 and 2100 m altitude.

Additional specimens examined—CHINA. YUNNAN: Dali Prefecture, Heqing County, Xiyi Town, Beiya Village, alt. 1800–1900 m, 26 August 2018, Jian-Wei Liu 1207 (HKAS 122950). Dali Prefecture, Heqing County, Xiyi Town, Beiya Village, alt. 1800–1900 m, 28 August 2018, Jian-Wei Liu 1281 (HKAS 122955). Dali Prefecture, Binchuan County, Jizushan Town, Shanqiansi Village, in forests with *Pinus yunnanensis*, alt. 2000 m, 11 August 2011, Ting Guo 438 (HKAS 71334).

Notes—Our phylogenetic analysis showed that *T. squamosus* shares, with high support, a most recent common ancestor with *T. fujisanensis*, *T. imbricatus*, *T. kauffmanii* and *T. parvisporus* (Figure 1). The large deltoid scales on pileal surface are a synapomorphy for these five species and appear to have evolved independently in gomphoid fungi.

***Turbinellus szechwanensis*** (R.H. Petersen) Xue-Ping Fan & Zhu L. Yang, **comb. nov.** (Figure 4 and Figure 5).

MycoBank: MB 843943

Basionym—*Gomphus szechwanensis* R.H. Petersen, Nova Hedwigia 21: 102 (1972) [“1971”].

Epitype—CHINA. SICHUAN: Garzê Prefecture, Kangding County, Yala Township, Mugecuo Scenic Spot, 9 September 2016, Bang Feng 130 (HKAS 99484, here designated! Genbank OQ858484/OQ858556/OQ847641).

Description—Basidioma 14–25 (–30) cm tall; unipileate; tapering downward. Pileus up to 17 cm in diam.; funnel-shaped; surface orange-ochre to brownish orange; pellicle-like. Hymenium surface clay colored; strongly wrinkled; irregularly reticulate and extending longitudinally to the upper stipe; dense. Stipe approximately cylindrical; rounded at base; hollow; upper part off-white and becoming reddish toward the base; glabrous above the base; context white.

Basidiospores 16–19.5 (–20) × 7.5–11 µm; oblong and inequilateral in side view with distinct suprahilar depression; ellipsoid in ventral view; non-amyloid; dull ochre in KOH; ornamentation of very coarse ridges or warts. Basidia 85–110 × 10–13 µm; clavate; generally 2–4-spored. Hymenial cystidia absent. Pileipellis composed of profusely inflated hyphae (8–21 μm in diam.), mostly with ampulliform swellings and little uninflated hyphae; simple branched; strictly parallel arranged toward pileal context; gloeoplerous hyphae abundant. Clamp connections absent in all parts of basidioma.

Habitat and locality—Gregarious in forests of *Abies*, *Picea*, *Pinus densata* and *Quercus semicarpifolia* in Southwest and central China at (2400–) 3200–4000 m altitude.

Specimens examined—CHINA. SICHUAN: Garzê Prefecture, Kangding County, locality unknown, 1894, M. Farges (Herb. Patouillard, HF, type of *G. szechwanensis*). Garzê Prefecture, Daofu County, Geka Township, 17 July 2014, Bang Feng 1598 (HKAS 93982). Garzê Prefecture, Xiangcheng County, Great Snow Mountain, in forests with *Abies* and *Rhododendron*, alt. 4000 m, Li-Song Wang 942 (HKAS 7871). HUBEI: Yichang City, Xingshan County, Shennongding Nature Reserve, 17 July 2012, Jiao Qin 569 (HKAS 77970). Yichang City, Xingshan County, Shennongding Nature Reserve, alt. 2400 m, 18 July 2012, Jiao Qin 586 (HKAS 77988). GANSU: Gannan Prefecture, Zhouqu County, Shatan Forest Park, in forests with *Abies*, 16 August 2012, Xue-Tai Zhu 730 (HKAS 76579). YUNNAN: Diqing Prefecture, Deqin County, Baima Snow Mountain, alt. 3700 m, Xing-Jiang Li 1142 (HKAS 8159). Diqing Prefecture, Xianggelila City, Xiaozhongdian Town, alt. 3500 m, 3 September 2018, Jian-Wei Liu 1597 (HKAS 122959). Diqing Prefecture, Xianggelila City, Xiaozhongdian Town, in forests with *Abies*, 2 September 2013, Bang Feng 1496 (HKAS 82602). Diqing Prefecture, Xianggelila City, Zhongdian Town, Bitahai Natural Reserve, in forests with *Picea*, alt. 3628 m, 3 September 2013, Bang Feng 1508 (HKAS 82614). Lijiang City, Yulong County, Alpine Garden, alt. 3525 m, 28 August 2019, Jian-Wei Liu 1711 (HKAS 122960). Lijiang City, Yulong County, Yulong Snow Mountain, in forests with *Pinus densata*, alt. 3231 m, 12 July 2010, Xue-Tai Zhu 24 (HKAS 68200). TIBET: Bomi County, on the way from Bomi to 24K, alt. 3257 m, 26 July 2017, Si-Peng Jian 46 (HKAS 101035). Bomi County, on the way from Bomi to Linzhi, alt. 2400 m, 18 July 2019, Zhu-Liang Yang 6201 (HKAS 106813). Gongbujiangda County, Niangdang Village, in forests with *Abies*, alt. 3812 m, 30 July 2014, Bang Feng 1656 (HKAS 94040). Rikaze City, Nielamu County, alt. 2400–2700 m, 3 September 2006, Jun-Feng Liang 613 (HKAS 51324). Linzhi County, National Highway 318, 757 m of northeast of Lulang Military Station, in forests of *Picea*, 29°49’ N, 94°44’ E, 22 July 2019, Geng-Shen Wang 511 (HKAS 116225). Linzhi County, Lulang Town, in forests of *Quercus semicarpifolia*, 29°58’ N, 94°51’ E, 18 July 2019, Geng-Shen Wang 436 (HKAS 116137).

Notes—*Gomphus floccosus* (Schwein.) Singer, under the name of *Cantharellus floccosus* Schwein., was first reported in China by Patouillard (1895), based on a collection made from Tchen-KéouTin (currently called Kangding) by M. Farges in 1894. This collection was treated as a new species by Petersen [6] and Zang et al. [59], namely *Gomphus szechwanensis*. Giachini [1] and Giachini and Castellano [60] synonymized *G. szechwanensis* under *T. floccosus*, it was originally described from North America, based on morphological analysis. However, our morphological and molecular data indicated that it is a species of *Turbinellus* and indeed differs from *T. floccosus* (Figure 1, Figure 4 and Figure 5); thus, a new combination was proposed. In order to fix the concept of the species, an epitype for the species was selected in this paper.

As mentioned by Petersen [6], *T. szechwanensis* is characterized by its larger basidioma exceeding 30 cm in height, broader pileus up to 17 cm in diam., pellicle-like surface of the pileus and coarsely decorated larger basidiospores. *Turbinellus floccosus* differs from this species by its significantly smaller basidiospores (10–16 × 5–8 µm in Arora [61]; 11.5–14.5 × 7–8 µm in Bessette et al. [62]). This species is closely related to *T. verrucosus* in the phylogenetic analysis (Figure 1), but the latter has a smaller basidioma less than 7 cm tall, a narrower pileus (2–4.5 cm in diam.) with verrucous scales and smaller basidiospores measuring 13–15.5 × 6–8 µm. Ecologically, *T. szechwanensis* mainly scatters in forests of *Abies*, *Picea* and *Pinus densata* at 3200–4000 m altitude. This is in contrast to several other species that occur at lower elevations, such as *T. verrucosus*, *T. squamosus*, *T. solidus* and so on. To date, only *T. szechwanensis* and *T. flavidus* of the genus have been founded in central parts of China.

**Figure 15:**
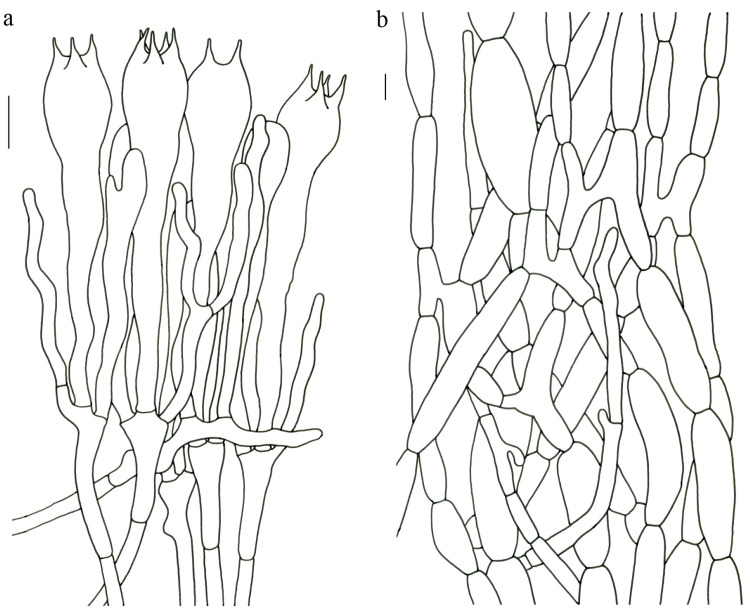
Microscopic features of *Turbinellus tomentosipes* (Type, HKAS 113156). (**a**) Hymenium and subhymenium; (**b**) Longitudinal section of pileipellis. Bars: (**a**)–(**b**) = 10 µm.

***Turbinellus tomentosipes*** Xue-Ping Fan & Zhu L. Yang, **sp. nov.** (Figure 4, Figure 5 and Figure 15).

MycoBank: MB 843965

Etymology—From the Latin tomentosipes, referring to the stipe of the new species covered with long tomentum.

Holotypus—CHINA. YUNNAN: Kunming City, Panlong District, Wild Duck Lake Park, alt. 2000–2200 m, 11 August 2020, Fei-Fei Liu 251 (HKAS 113156; Genbank OQ858453/OQ858521/OQ847611).

Diagnosis—*Turbinellus tomentosipes* is similar to *T. solidus*, but the former possesses a larger basidioma up to 15 cm tall, a densely tomentose stipe and profusely inflated hyphae in stipitipellis. The latter has a smaller basidioma less than 10 cm tall, a glabrous stipe and little inflated hyphae in stipitipellis.

Description—Basidioma 5–15 cm tall; unipileate or bipileate; tapering downward. Pileus 2–8 cm in diam.; funnel-shaped; depressed at center; surface orange-yellow (5A7–8) when young and faint yellow (1A2–4) with age; rough and sparsely covered with minute appressed scales; scales more or less radially arranged and usually darker than the pileal surface; appearing glutinous; pileal margin extensional and undulate. Hymenium surface white (1A1) to faint yellow (1A2–4) or creamy orange (4A2–3); weakly wrinkled; longitudinal ridges extending to the upper stipe; branching irregularly upwards; sparse; thin to 0.2 cm. Stipe about 3–6 cm long, 0.7–2 cm in diam.; approximately cylindrical; solid; creamy orange (4A3) when young and creamy (1A3) with age; densely covered with long creamy yellow (4A2) tomentum; context pliable; thin to 0.2–0.4 cm; white (1A1) to creamy (1A2); unchanging on exposure; basal mycelium white (1A1).

Basidiospores [63/3/3] 11–14 × 5.5–7.5 (–8) µm; Q = (1.53–) 1.58–2.15 (–2.36); Qm = 1.91 ± 0.17; oblong and inequilateral in side view with distinct suprahilar depression; ellipsoid in ventral view; non-amyloid; light yellowish in KOH; ornamentation of very coarse ridges or warts. Basidia 50–75 × 8–13.5 µm; clavate, 2–5-spored; sterigmata 4–7 µm long. Hymenial cystidia absent. Pileipellis composed of profusely inflated hyphae (7–24 μm in diam.); mostly with ampulliform swellings and little uninflated hyphae (3–6 μm in diam.); simple branched; constricted at septa; repent; more or less parallel arranged toward the pileal context; gloeoplerous hyphae abundant; yellow in KOH. Stipitipellis composed of some basidia-like cells; profusely uninflated hyphae (2–6 μm in diam.) and some inflated hyphae (7–12 μm in diam.); gloeoplerous hyphae abundant; yellow in KOH. Pileus and stipe context composed of simple branched hyphae; approximately parallel; hyaline to yellow in KOH. Hymenial trama composed of subparallel to interwoven, undifferentiated and narrow hyphae. Clamp connections absent in all parts of basidioma.

Habitat and distribution—Scattered in subtropical forests dominated by plants of *Pinus yunnanensis*, *Quercus* and *Rhododendron decorum*; fruiting from June to August in southwestern China (Yunnan Province) between 1000 and 2200 m altitude.

Additional specimens examined—CHINA. YUNNAN: Chuxiong Prefecture, Lufeng City, Heijing Town, in broad-leaved forests, alt. 1028 m, 10 July 2014, Xiao-Bin Liu 419 (HKAS 87066). Dali Prefecture, Binchuan County, Jizushan Town, Shanqiansi Village, 23°45′ N, 105°12′ E, alt. 1350 m, 11 August 2011, Ting Guo 436 (HKAS 71332). Dali Prefecture, Binchuan County, Jizushan Town, Siqian Village, in forests of *Pinus yunnanensis*, *Quercus* and *Rhododendron decorum*, 25°56′ N, 100°23′ E, alt. 1988 m, 11 August 2011, Li-Ping Tang 1541 (HKAS 70002). Wenshan Prefecture, Qiubei County, Jinping Town, Xiangqi Village, in forests with Fagaceae and *Pinus yunnanensis*, alt. 1570 m, 10 August 2014, Pan-Meng Wang 141 (HKAS 95222).

Notes—*Turbinellus tomentosipes* is similar to *T. solidus*, but the latter possesses a smaller basidioma, a glabrous stipe and filamentous hyphae in stipitipellis. Geographically, *T. tomentosipes* occurs in northern parts of Yunnan, while *T. solidus* fruits in its southern parts.

**Figure 16:**
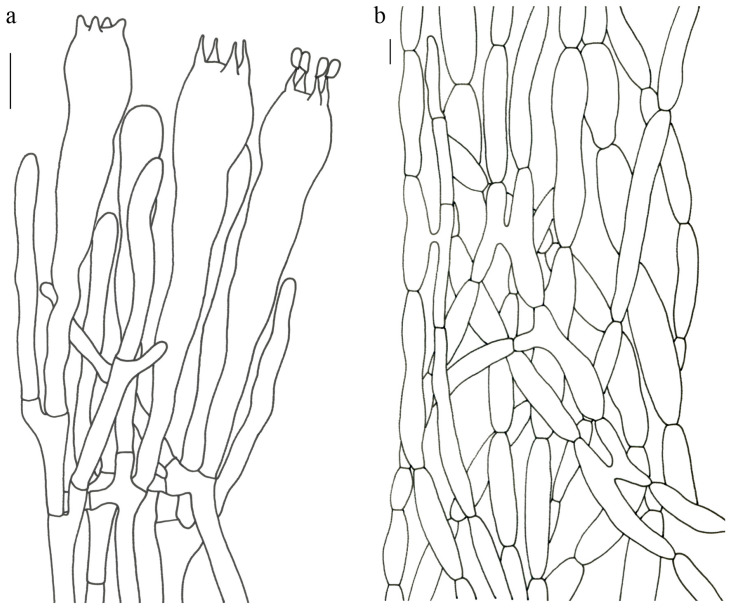
Microscopic features of *Turbinellus verrucosus* (Type, HKAS 74860). (**a**) Hymenium and subhymenium; (**b**) Longitudinal section of pileipellis. Bars: (**a**)–(**b**) = 10 µm.

***Turbinellus verrucosus*** Xue-Ping Fan & Zhu L. Yang, **sp. nov.** (Figure 4, Figure 5 and Figure 16).

MycoBank: MB 843966

Etymology—From the Latin adjective, verrucosus, referring to the pileus densely covered with verrucose protuberances of the new species.

Holotypus—CHINA. YUNNAN: Nujiang Prefecture, Lushui City, Laowo Township, Chongren Village, mainly in forests with *Pinus yunnanensis*, supplemented by Fagaceae and Ericaceae, alt. 1700–1800 m, 7 August 2011, Gang Wu 546 (HKAS 74860; Genbank OQ858538).

Diagnosis—*Turbinellus verrucosus* is similar to *T. solidus* in having small basidioma, orange pileus and small basidiospores. However, the former has a pileus with densely verrucous protuberances, a hollow stipe and little inflated hyphae in stipitipellis. The latter has a pileus with sparsely appressed scales, a solid stipe and plenty of inflated hyphae in stipitipellis.

Description—Basidioma 4–6.5 cm tall; unipileate; tapering downward. Pileus 2.2–4.5 cm in diam.; funnel-shaped; depressed at center; surface orange (5A5–8) to dark red (7C6); rough and densely covered with verrucous protuberances; protuberances more or less radially arranged; pileal margin in-rolled and nearly entire. Hymenium surface off-white (1A1) to light yellow (2A4) or creamy orange (5A2); strongly wrinkled; longitudinally reticulate and extending to the stipe base; dense; thick to 0.2–0.4 cm; not reaching creamy yellow (4A3) to orange (5A6); margin upwards. Stipe about 1.5–3.5 cm long; 0.5–0.8 cm in diam.; approximately cylindrical; occasionally dilating obconically into the pileus; hollow; white (1A1) to creamy (2A3) or creamy orange (5A3); glabrous above the base; context pliable; thin to 0.1–0.2 cm; white (1A1); unchanging on exposure; basal mycelium white (1A1).

Basidiospores [61/3/3] 13–15.5 (–16) × 6–8 (–8.5) µm; Q = (1.69–) 1.86–2.25 (–2.33); Qm = 2.01 ± 0.14; oblong to cylindrical and inequilateral in side view with distinct suprahilar depression; ellipsoid in ventral view; non-amyloid, light yellowish in KOH; ornamentation moderately verrucose with coarse warts. Basidia 70–95 × 11–16 µm; clavate; generally 4-spored; sterigmata 5–10 µm long. Hymenial cystidia absent. Pileipellis composed of profusely inflated hyphae (7–19 μm in diam.) mostly with ampulliform swellings and little uninflated hyphae (3–6 μm in diam.); simple branched; constricted at septa; repent; more or less parallel arranged toward the pileal context; gloeoplerous hyphae abundant; yellow in KOH. Stipitipellis composed of some basidia-like cells; plenty of uninflated hyphae (2–6 μm in diam.) and little inflated hyphae (7–10 μm in diam.); gloeoplerous hyphae abundant; yellow in KOH. Pileus and stipe context composed of simple branched hyphae; approximately parallel; hyaline to yellow in KOH. Hymenial trama composed of subparallel to interwoven, undifferentiated and narrow hyphae (3–6 μm in diam.). Clamp connections absent in all parts of basidioma.

Habitat and distribution—Scattered in subtropical forests with *Pinus*; fruiting from June to August in southwestern China (Yunnan Province) between 1250 and 2100 m altitude.

Additional specimens examined: CHINA. YUNNAN: Baoshan City, Longyang District, Lujiang Township, Pumanshao, in the mixed coniferous broad-leaved forests, alt. 2033 m, 23 March 2014, Xiao-Bin Liu 708 (HKAS 87257). Baoshan City, Changning County, Wojiaodi Village, alt. 1784 m, 24 July 2009, Li-Ping Tang 931 (HKAS 56888). Dali Prefecture, Heqing County, Xiyi Town, Beiya Village, 26 August 2018, Jian-Wei Liu 1180 (HKAS 122949). Puer City, Lancang County, Donghui Township, in forests with *Pinus kesiya*, alt. 1260 m, 29 August 2017, Zhu-Liang Yang 5996 (HKAS 101185). Puer City, Lancang County, Menglang Township, Zhazhai Village, 21 August 2016, Jian-Wei Liu 50 (HKAS 97554). Puer City, Jiangcheng County, 29 July 2008, Li-Ping Tang 511 (HKAS 54742). Sipsongpanna, Jinghong City, alt. 1400 m, 1 August 2008, Xi-Hui Du 19 (HKAS 55476).

Notes—*Turbinellus verrucosus* is distinguished by the combination characters of the small basidioma, orange to orange-red pileus densely covered with verrucous protuberances and hollow stipe. This species can be easily confused with *T. floccosus*, native to North America, due to their similar appearance of basidiomata. However, *T. floccosus* possesses a significantly larger basidioma (5–20 cm), a larger pileus (3–15 cm) and a thicker stipe (3–10 × 1–3 cm) [61]. Moreover, our molecular data resolved *T. floccosus* as a lineage independent of those *T. floccosus*-like fungi in China (Figure 1).

***Turbinellus yunnanensis*** (R.H. Petersen & M. Zang) Xue-Ping Fan & Zhu L. Yang, **comb. nov.** (Figure 4 and Figure 5).

MycoBank: MB 843944

Basionym—*Gomphus yunnanensis* R.H. Petersen and M. Zang, in Zang, Li and Xi, Fungi of the Hengduan Mountains: 181 (1996).

Description—Basidioma up to 12 cm tall; unipileate; tapering downward. Pileus up to 7 cm in diam.; funnel-shaped; depressed at center; surface orange to orange-red; sparsely covered with minute appressed scales; pileal margin undulate to entire. Hymenium surface orange-red to reddish; wrinkled; irregularly reticulate and extending to the upper stipe; sparse. Stipe 1–4 cm long; 1–1.7 cm in diam.; subcylindrical; hollow; orange-red to reddish; glabrous above the base; context white.

Basidiospores 13–17 × 6.5–7.5 µm; oblong and inequilateral in side view with distinct suprahilar depression; oblong in ventral view; non-amyloid; ornamentation of very densely coarse ridges or warts. Hymenial cystidia absent. Gloeoplerous hyphae abundant in pileipellis, stipitipellis and context. Clamp connections absent in all parts of basidioma.

Habitat and locality—Gregarious in forests of *Abies* and *Picea*; fruiting from summer to autumn in southwestern China between 2800 and 3200 m altitude.

Specimens examined—CHINA. YUNNAN: Lijiang City, Yulong County, Yulong Snow Mountain, in forests of *Abies* and *Picea*, alt. 3000–3200 m, 3 September 1986, R.H. Petersen 56,349 (HKAS 18142, holotype of *Gomphus yunnanensis*; Genbank OQ858460/OQ858528/OQ847616). Lijiang City, Yulong County, Yulong Snow Mountain, in forests of *Picea*, alt. 3000 m, 6 September 1986, R.H. Petersen 56,818 (HKAS 18145). Diqing Prefecture, Xianggelila City, Haba Snow Mountain, alt. 2858 m, 1 October 2007, Bang Feng 195 (HKAS 52916). Lijiang City, Yulong County, Yulong Snow Mountain, in forests of *Pinus densata*, alt. 3048 m, 18 August 2013, Yang-Yang Cui 115 (HKAS 79785). Lijiang City, Yulong County, Alpine Garden, 26 September 2019, Jian-Wei Liu 2143 (HKAS 122962). Lijiang City, Yulong County, Lijiang Observatory, in forests of *Picea*, *Pinus* and *Quercus*, alt. 3200 m, 17 August 2014, Qi Zhao 2147 (HKAS 87913).

Notes—*Turbinellus yunnanensis* was once placed in the genus *Gomphus* [59]. Our morphological and molecular data indicated that it is a species of *Turbinellus* (Figure 1, Figure 4 and Figure 5); thus, a new combination was made. *Turbinellus yunnanensis* is characterized by its reddish basidioma, orangish pileus, hollow stipe and medium-sized basidiospores (13–17 × 6.5–7.5 µm). *Turbinellus yunnanensis* is similar to *T. longistipes* and *T. solidus* in having orange-yellow to orange pileus with minute appressed scales. However, *T. yunnanensis* differs from *T. longistipes* in its shorter cylindrical stipe and smaller basidiospores from *T. solidus* with its reddish basidioma and hollow stipe. Furthermore, *T. yunnanensis* is distributed in forests of *Abies*, *Picea*, *Pinus densata* and *Quercus* at 2800–3200 m altitude, while *T. longistipes* occurs in forests of *Abies*, *Picea*, *Pinus densata* and *Quercus semicarpifolia* at 3200–3600 m altitude in the northern parts of Yunnan. *T. solidus* occurs in forests of Fagaceae and *Pinus* at 1300–1600 m altitude in the area’s south.

## 4. Discussion

### 4.1. Phylogeny and Diversity of Gomphoid Fungi

*Gomphus* harbors conspicuous members of Gomphaceae and are often confused with other genera in the family. Recent analyses [3,63,64,65] indicated that *Gomphus,* in its broader sense, constitutes a paraphyletic group of fungi with *Ramaria* sensu lato. To maintain the monophyly of *Gomphus*, some species were transferred to *Gloeocantharellus*, and *Turbinellus* was resurrected [1,3]. *Gomphus* and *Turbinellus*, as defined here, are monophyletic. This classification is consistent with some previous studies [1,3]. Our phylogenetic analysis supports a sister relationship between *Gomphus* and *Turbinellus*, contrasting with those studies in which *Turbinellus*, together with two or more genera of Gomphaceae, were found to be sisters to either *Gomphus* alone or *Gomphus* together with several species of *Ramaria* [2,63,64,65]. The previous definition of gomphoid fungi [1], including *Gloeocantharellus*, turned out to be unnatural. Our analyses supported the monophyly of the currently defined gomphoid fungi in the narrower sense, only including *Gomphus* and *Turbinellus*.

Most gomphoid species are known from the northern hemisphere, especially North America, where Corner [57] and Petersen [6] considered to be the diversity center of this group. However, according to the latest studies [1,66,67], only four species of this group, namely *G. clavatus*, *G. ludovicianus*, *T. floccosus* and *T. kauffmanii*, have been recognized in North America. Our study first included a large number of Asian samples and clarified the phylogenetic relationships of most known species of gomphoid fungi. Based on our study and previous ones [1,66,67], 23 gomphoid species are currently recognized from Africa, Asia, Europe and North America. Our study indicated that gomphoid species in Asia are abundant and different from those in the other continents. Except for the widely distributed species *G. clavatus*, *G. crassipes* and *T. floccosus*, which have been reported in two or three continents, the remaining species are endemic either to Africa, including *G. brunneus*, or to North America, including *G. ludovicianus* and *T. kauffmanii* or to East Asia (or China), including *G. matijun*, *G. orientalis*, *T. fujisanensis*, *T. szechwanensis*, *T. yunnanensis* and the new species described here.

*Turbinellus floccosus*, easily identified due to the funnel-shape and orange color system of its basidioma, was supposed to be widely distributed in the northern hemisphere [1,57,68]. However, our study indicated that its occurrence in China or in East Asia needs to be verified. Gomphoid fungi in southwestern China, including the Yunnan-Guizhou Plateau, the Hengduan Mountains region and the Qinghai-Tibet Plateau, show a very high diversity with at least 14 species, accounting for 61% of the total, thus indicating another hotspot of gomphoid fungi in the northern hemisphere. However, geographic configuration of the whole group, as well as the comprehensive phylogenetic relationship, remain unresolved. Increasing taxon sampling in different regions and enlarging the molecular information are essential for further studies.

### 4.2. Trade-Offs among Traits Related to Reproduction in Evolution

Our results showed a close relationship between spore size and spore shape in gomphoid fungi in the narrower sense. When the spores expand in size, they become more spherical, and these changes occurred almost simultaneously (Figure 3D,K,L), suggesting that the spore size and spore shape are evolutionarily synchronized. That could be due to internal physiological or anatomical constraints, making it difficult to produce both large and narrow spores [13]. That argument is also supported by the fact that spore expansion is the result of simultaneous expansion of both length and width based on our analysis of character evolution, rather than simply length or width extension (Figure S1A–C). As for the tendency for spore evolution to be larger and more spherical beginning in the Cenozoic era, it could be a survivability trade-off driven by global cooling and global aridity [69]. Large spores which are spherical with smaller surface to the volume ratio, compared to small and narrow spores, will contain more water and more nutrients to fight the cold and avoid large amounts of water evaporation [13,26], which can be essential for successful germination [70]. The nutritional mode also accounted for a significant part of the fluctuation in spore size and shape [12,13], with ectomycorrhizal species producing significantly large and spherical spores, in line with the fact that ectomycorrhizal spores need to survive until conditions for root colonization are suitable.

Species producing large spores might need large fruit bodies to produce a high number of spores [13,71,72]. However, the fruit body size of gomphoid fungi s. str. did not vary with spore size in that correlation, as expected during the Mesozoic, when gomphoid species had relative larger fruit bodies with smaller spores (Figure 3B,J,K). This trend has surprisingly reversed in the Cenozoic, when gomphoid fungi s. str. have relative smaller fruit bodies with larger spores than their Mesozoic ancestors (Figure 3B,F,G). An explanation for gomphoid species with smaller fruit bodies after the Mesozoic is the need to adapt to aridification, especially during the late Eocene and beyond, when it was generally more arid than before due to global cooling [73,74,75]. Water supply is crucial for fungal growth [76], as mentioned by Halbwachs et al. [20]; many macrofungi experience considerable dwarfing in dry years. However, the decrease in fruit body size was not followed by stasis or acceleration; rather, fruit body size enlarged through the Quaternary (Figure 3F,J). Fruit body size increase should not be the result of spore size expansion; there were no lineage-consistent changes in fruit bodies and spores of phylogenetic character mapping (Figure S1E,F). However, this variation is in accordance with water availability, as shown by Halbwachs et al. [20]. As the Himalaya orogeny entered its late-stage, the East Asian monsoon was strengthened and the precipitation in the collision zone of the Indo-Asian plate increased, allowing the fruit body to enlarge. A larger fruit body may be a greater buffer against cold stress, which was also beneficial for survival during periods of sharply global cooling.

### 4.3. Larger Spores Were More Important Than Larger Fruit-Bodies

Based on analyses of characters combined with their evolution, we found that Cenozoic and present gomphoid fungi s. str. had larger spores relative to their fruit body size than those of Mesozoic taxa (Figure 3B,G), and the spores showed a trend of rapid enlargement in the Cenozoic excluding the late Eocene (Figure 3G,K). When expressed as a phylogenetic sporulation quotient (PSQ), spore sizes of Cenozoic taxa developed more rapidly than those of Mesozoic taxa, and high rates continued through the Quaternary (Figure 2A). All data available suggested that spores have expanded dramatically after the end-Cretaceous extinction, as a critical innovation enhancing spore survival and germination through time and space, and thereby insuring their persistence and ecological success. Conversely, the fruit body size of Paleogene species contracted rapidly and continued into the Neogene (Figure 3F,J), although briefly enlarged during the middle Eocene, when there was an episode of Eocene climatic optimum with humid conditions [69,77,78]. They were essentially smaller than their sizes in the Mesozoic era (Figure 3F,J), which could have meant larger fruit body numbers [12]. However, our study found no direct evidence that these innovations after the end-Cretaceous extinction contributed to the diversification of gomphoid fungi s. str. Rates of speciation and net diversification of this group increased steadily through time (Figure 2C), which were also consistent with LTT plots (Figure 2D). There was no rate shift within the gomphoid fungi s. str., suggesting no radiative evolution event occurred. That is, these innovations did not lead to rapid speciation after the end-Cretaceous extinction.

As the Mesozoic transitioned to the Cenozoic, the mode of spore and fruit body evolution shifted. Relative spore size greatly increased, in both average and variance, but the fruit body size continued to decrease, signaling a new regime in which spore size enlargements were more paramount than those in fruit body size. Greater fruit body numbers were also more advantageous than those in large fruit bodies. The growth of spores was not only in size, but also in shape, i.e., being more spherical, thus greatly increasing capacity of water retention. The rate of change in PSQ was stable across the Mesozoic but increased dramatically near the beginning of the Cenozoic or immediately after the end-Cretaceous extinction (Figure 2A); this preceded the increase in the rate of change in fruit body size (Figure 2B). This suggests that selection acted differently on spores and fruit body size, and, at the very least, relatively large fruit bodies were not necessary for gomphoid fungi s. str. after the end-Cretaceous extinction. Smaller fruit bodies, alternatively meaning a greater number of fruit bodies, were considered to have an advantage when colonizing patchy niches as extinction survivors, with option for a finer-grained response to reproductive investment over larger fruiting species and reducing the risk of demising before sporulation [8,12]. The majority of branches exhibited faster rates of fruit body size increase in the late Miocene. This reached its maximum in the Quaternary (Figure 2B), during which fruit body size continued to enlarge (Figure 3F,J), thus indicating that the larger fruit body was more likely driven by the sharply global cooling and the strengthening of the East Asian monsoon.

## Data Availability

Not applicable.

## Supplementary Materials

The following supporting information can be downloaded at https://www.mdpi.com/article/10.3390/jof9060626/s1, Figure S1: (A–C) Log10(spore size), log10(spore width) and log10(spore length) average through time per 10-million-year bins. (D–G) Ancestral state reconstructions of log10(PSQ), log10(fruit body size), log10(spore size) and log10(Qm) mapped onto a phylogenetic tree. Table S1: Taxa sampled in phylogenetic analysis with information related to GenBank accession numbers, locality, references, taxonomy and voucher information [3,64,66,79,80,81,82,83,84,85]. Table S2: Characters used for gomphoid fungi. Table S3: Four secondary calibration ages for divergence time estimation. Numbers in the first column correspond to those in Figure 2A.
