# Supplementary figures and images for: Phylogeny, Taxonomy and Evolutionary Trade-Offs in Reproductive Traits of Gomphoid Fungi (Gomphaceae, Gomphales)
Source: J Fungi (Basel). 2023 May 29;9(6):626. doi: 10.3390/jof9060626 (PMC10301500; doi:10.3390/jof9060626)

**A**

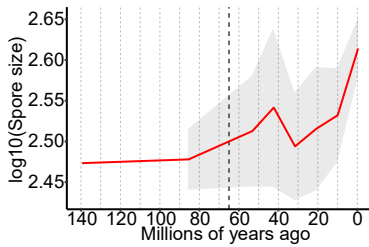

**B**

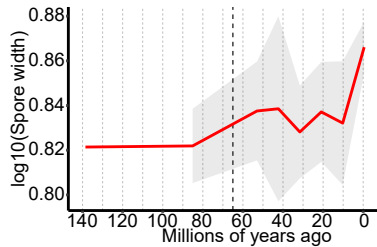

**C**

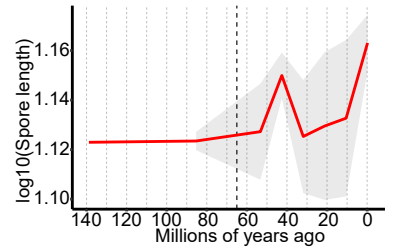

D

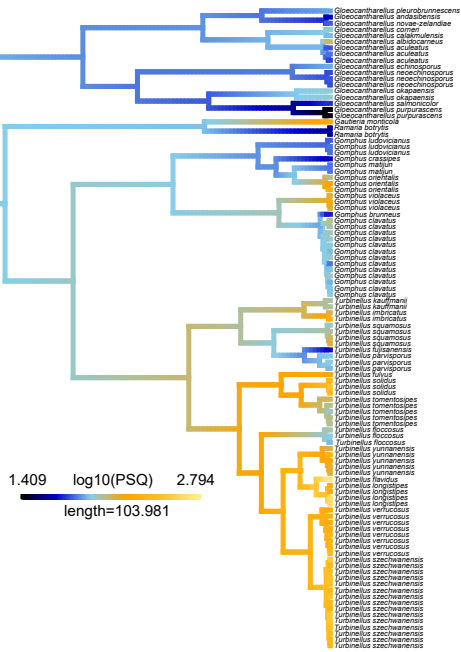

# E

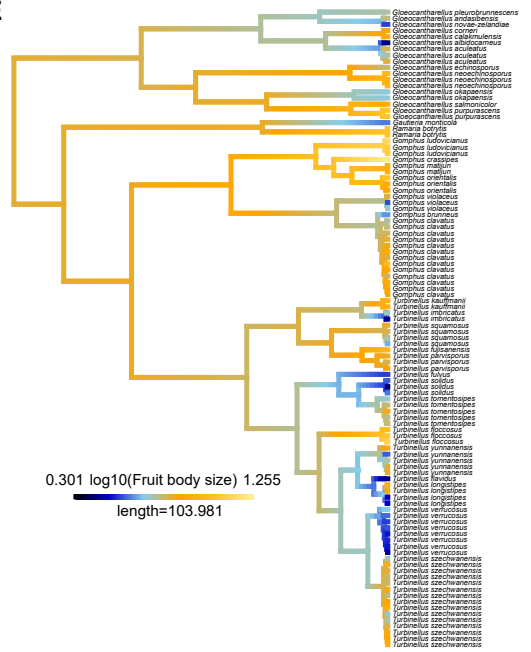

**F**

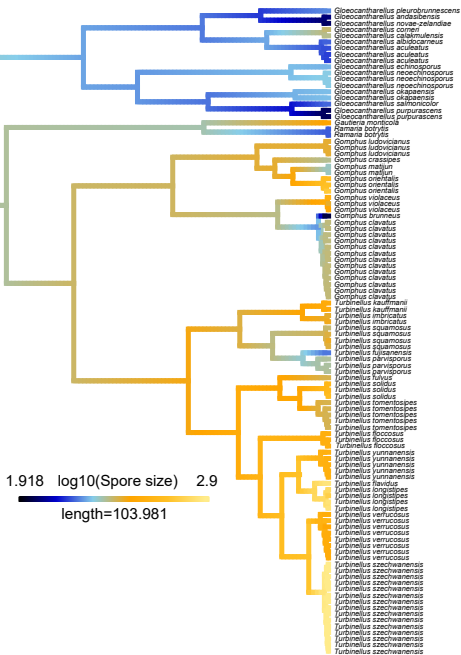

**G**

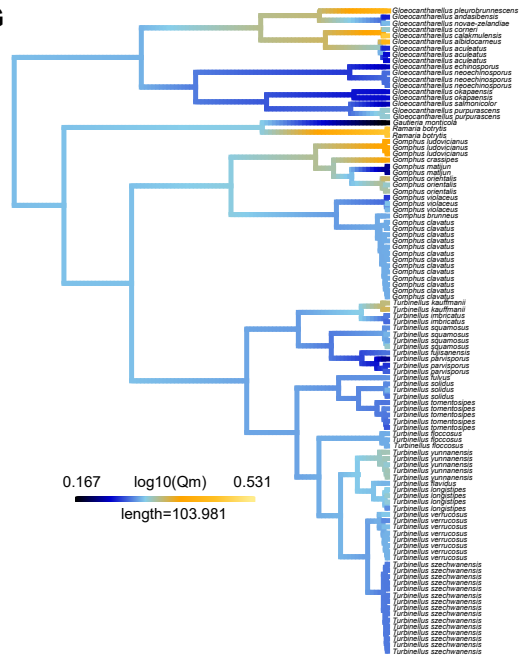

Supplement: Supplementary file 1 [file jof-09-00626-s001.zip › Figure S1.pdf]
